# The effects of emergency medical service work on the psychological, physical, and social well-being of ambulance personnel: a systematic review of qualitative research

**DOI:** 10.1186/s12888-020-02752-4

**Published:** 2020-07-03

**Authors:** Sharon Lawn, Louise Roberts, Eileen Willis, Leah Couzner, Leila Mohammadi, Elizabeth Goble

**Affiliations:** 1 College of Medicine and Public Health, Flinders University, GPO Box 2100, Adelaide, SA 5001 Australia; 2 College of Nursing and Health Sciences, Flinders University, GPO Box 2100, Adelaide, SA 5001 Australia

**Keywords:** Paramedics, Ambulance officers, Ambulance volunteers, Call-takers, Mental health, Well-being, Organisational culture, Vocational health, Qualitative

## Abstract

**Background:**

High rates of mental distress, mental illness, and the associated physical effects of psychological injury experienced by ambulance personnel has been widely reported in quantitative research. However, there is limited understanding of how the nature of ambulance work contributes to this problem, the significant large toll that emergency medical response takes on the individual, and particularly about late and cumulative development of work-related distress among this first responder workforce.

**Methods:**

This study examined peer-reviewed qualitative research published from 2000 to 2018 to outline the effect of emergency medical response work on the psychological, psychosocial, and physical health of paramedics, ambulance officers, ambulance volunteers, and call-takers. Databases searched included: Ovid Medline, CINAHL, Ovid EMcare, PsychInfo and Scopus. The systematic review was organised around five key areas: impact of the work on psychological wellbeing; impact of psychological stress on physical wellbeing; how work-related well-being needs were articulated; effects of workflow and the nature of the work on well-being; and, effects of organisational structures on psychological and physical well-being.

**Results:**

Thirty-nine articles met the eligibility criteria. Several factors present in the day-to-day work of ambulance personnel, and in how organisational management acknowledge and respond, were identified as being significant and contributing to mental health and well-being, or increasing the risk for developing conditions such as PTSD, depression, and anxiety. Ambulance personnel articulated their well-being needs across four key areas: organisational support; informal support; use of humour; and individual mechanisms to cope such as detachment and external supports.

**Conclusions:**

Interactions between critical incidents and workplace culture and demands have an overwhelming impact on the psychological, physical and social well-being of ambulance personnel. These include day-to-day managerial actions and responses, the impact of shift work, poorly-managed rosters, and long hours of work with little time between for recovery. Mental health issues result from exposure to traumatic events, and the way managers and peers respond to worker distress. Ambulance personnel suffering from work-related stress feel abandoned by peers, management, and the service, during illness, in return-to-work, and post-retirement. Policy, programmes and interventions, and education need to occur at an individual, peer, organisational, and government level.

## Background

Ambulance personnel are essential first responders in the community. Across different countries and jurisdictions, they are known by a variety of terms such as paramedics, emergency medical technician, emergency medical personnel, emergency dispatch personnel and call-takers. Their role is to directly provide or coordinate the communication of response for out-of-hospital or pre-hospital emergency medical care in the community. However, they are arguably ‘the forgotten profession’ within the healthcare system. Their contribution to the health and wellbeing of the community and to healthcare is overshadowed by more dominant dialogues and debates about community services, acute care and hospital emergency department tensions and resource demands [1–4].

In Australia, the nature of ambulance work, the uncontrolled and often unpredictable environments, the everyday experience of trauma, and the cumulative nature of that trauma all play a key role in the development and impact of mental distress and psychological injury [3–5]. In addition to the nature of the work, organisational and occupational factors such as workload, work demands, shift work, limited time for debriefing or downtime, the hierarchical nature of supervision, and the lack of recognition are clearly shown to have effects on the well-being of ambulance personnel that are as significant as, if not greater than, the nature of the work itself [3, 5, 6]. This paper examines the peer-reviewed qualitative research to outline the effect of emergency medical response work on the psychological, psychosocial, and physical health of paramedics, ambulance officers, ambulance volunteers, and call-takers.

A recent systematic review of 27 international studies [7] reported on 30,878 ambulance personnel and found estimated prevalence rates of 11% for post-traumatic stress (PTS), 15% for depression, 15% for anxiety, and 27% for general psychological distress among ambulance personnel. A broader Canadian study [8] of emergency response and correctional workers (5813 correctional workers, dispatchers, firefighters, paramedics, and police officers) showed that 44.5% screened positive for clinically significant symptoms of one or more diagnosable mental disorders; approximately four times higher than diagnosed rates for the general population at 10.1%. Of concern, these rates were noted to be higher than earlier studies and suggested the rate of anxiety among paramedics to be as high as 22%, with depression and suicidal ideation both at 10% [8]. Of added concern, under-reporting is thought to be a pervasive feature of this healthcare workforce [2, 5]. Apart from psychological impacts, a range of physical impacts resulting from the nature of ambulance work and exposure to occupational stress have also been reported. These include headaches, sleep disruption, muscular skeletal injuries, fatigue, dietary problems, weight gain and, in some cases, exposure to dangerous pathogens [9–11]. The research therefore suggests that rates of mental distress, mental illness, and the associated physical effects of psychological injury experienced by ambulance personnel demonstrate the large toll that emergency medical response takes on the individual.

Safe Work Australia [12], the Australian government statutory body established to develop national policy for work health and safety and workers’ compensation, reports that, although serious mental disorder claims from first responders account for only around 10% of all claims, they have significantly more impact on the individual than other claims. These impacts included extended time off work and significant compensation for ongoing care and support. Claims were almost five times longer than for other serious claims for all types of injuries and illnesses, and monetary payouts for serious mental disorder for first responders was almost double that of all payments [12]. These statistics demonstrate the high cost in monetary value, lost productivity, and lost personnel which is experienced by Ambulance Services personnel and other first responders due to the effect of mental distress and psychological injury [12].

The predominant use of measures of stress and psychological injury which focus on current symptoms make the longitudinal screening of personal mental health and wellbeing difficult and potentially miss important information about late and cumulative development of work-related distress. This mismatch may result in failure to recognise and address adverse longer-term impacts of the work itself. It may also result in failure to identify effective evidence-based prevention measures or to intervene early to prevent potentially higher rates and prevalence of psychological distress in this group [6].

Granter et al’s [13] recent study with ambulance services in England explored how emergency workers respond to the varied and multidimensional nature of their work and how this influences resilience and vulnerability to distress. Their study highlighted a cross-section of intense, high energy, and time-critical aspects of the work intermixed with a mundane, operational, and bureaucratic work life [13]. This mixture of high intensity and mundane work often created a difficult shift for paramedics’ mindset, with little respite or time for debriefing and dealing with administrative requirements during periods of intense emotions [13].

In Australia, and internationally, there has been extensive measurement of prevalence of this problem and increasing focus on policy development, service-based strategies, and programs for promoting mental health and wellbeing among ambulance personnel and other first responder groups. To date, quantitative study designs have provided the basis for these developments but with limited focus on the lived experience and qualitative evidence. Despite these initiatives and the existence of a broad range of organisational support services and programs, concern for the mental health of ambulance personnel continues to grow. This review aims to provide a stronger spotlight on the lived experience of ambulance personnel to identify the gaps in provision of support and care, and the challenges faced by those who experience psychological distress as a consequence of their everyday work.

## Methods

### Aim and research questions

The aim of this study is to provide a comprehensive review of relevant international peer-reviewed qualitative literature on the effects of emergency medical service work on the psychological, physical, and social well-being of ambulance personnel (paramedics, ambulance officers, ambulance volunteers, and call-takers). The Ambulance Employees Association of South Australia (AEASA) which commissioned this review were aware of the extensive quantitative research in this area but wanted to see what the qualitative research showed that might help to expand our understanding and better explain potential contributors to psychological, physical and social well-being status in ambulance personnel. In this sense, they were interested in answers to the following three broad research questions: What is the problem? What causes the problem? What is needed to address the problem?

The following questions guided this systematic review:

1. What impact does emergency service work have on the psychological well-being, and psycho-social health of paramedics, ambulance officers, ambulance volunteers, and ambulance call-takers?
2. What impact does the psychological stress linked to the workplace have on physical well-being for paramedics, ambulance officers, ambulance volunteers, and call-takers?
3. How do paramedics, ambulance officers, ambulance volunteers, and call-takers articulate their work-related well-being needs?
4. How are paramedics’, ambulance officers’, ambulance volunteers’, and call-takers’ mental health and well-being effected by workflow, the nature of work, and their changing roles?
5. What effect do organisational structures addressing respite, debriefing (both formal and informal) and workload have on paramedic, ambulance officers, and ambulance volunteers’ psychological and physical well-being?

Further questions about perceived stigma, help-seeking behaviours and how ambulance personnel identify and report signs of psychological distress related to their work were also explored for this systematic review. However, dedicated examination of these specific issues will be reported elsewhere.

### Search methods and screening

A systematic literature search was conducted to identify the relevant peer-reviewed literature using the PICO tool for qualitative research to identify key concepts pertinent to ‘participant problem (or population)’, ‘intervention’, ‘comparison or control’ and ‘outcome’ [14]. Research on the topic of this review is relatively young and has proliferated predominantly in the last two decades, with widespread professionalisation of the paramedic workforce via transfer of paramedic education to the university section in Australia, New Zealand and the UK around 2000, resulting in an increase in paramedic academics and subsequent research being conducted by them; therefore, only studies published since 2000 were included [15]. The main search was conducted in the Ovid Medline database, and incorporated both Subject headings (MeSH: Medical subject headings) and text words, and then translated into the PsycInfo, Ovid EMcare, CINAHL, and Scopus databases (executed in October 2018; see Appendix 1). Retrieved results of the database searches were then exported into Endnote for collation. A PRISMA diagram was used to report the search results and screening process. The titles and abstracts of all results were screened independently by two reviewers with a third reviewing any discrepancies, based on inclusion and exclusion criteria shown in Table 1. Full texts deemed eligible for inclusion based on their title and abstract were obtained for further screening.

**Table 1:** Inclusion and Exclusion Criteria for Peer-Reviewed Literature

Inclusion criteria	Exclusion criteria
● Paramedic/Emergency Medical Service EMS)-based populations including the following roles and terms: paramedic, ambulance personnel, community paramedic, intensive care paramedic, emergency medical technician, emergency medical personnel, emergency dispatch personnel, emergency call-takers, ambulance volunteers, out-of-hospital or pre-hospital paramedic	● Not specific to paramedic/EMS-based populations; focused on other ESFR populations (fire, police, state emergency services)
	● Studies focus on undergraduates studying to become Ambulance personnel
● Emergency service first responder (ESFR) studies in which data for paramedic populations is clearly articulated and distinguish from other ESFR populations	
	● Not available in the English language
● Interventions related to mental health, psychological wellbeing, work related stress, physical health	● Published prior to 2000
● Outcomes related to mental health, psychological wellbeing, work related stress, physical health	● Non-peer-reviewed literature
● Published in the English language	● Editorials, opinion pieces
● Systematic reviews (provided they reported at least one qualitative study)	● Quantitative data only reported
● Published January 1st 2000–2018	● Based on a single incident (e.g. disaster, terrorism) or focused on a specific case type/patient cohort (e.g. children, end-of-life care, CPR performance, Ebola, forensic)
● Peer-reviewed literature	
● Reported an empirical study	
● Other identified reviews (e.g., narrative, scoping, rapid)	
● Used qualitative data collection methods (for all, or some, components of the research)	

The review was registered with PROSPERO (Registration No.CRD42019117397).

### Data extraction and synthesis

The following data was extracted from each study: aim and methodology; sample characteristics; data collection methods; data analysis methods; and study limitations (see Appendix 2). The findings of each study were extracted and collated according to the focus questions (see Appendix 3 and 4). This enabled a thematic narrative synthesis to be undertaken in which commonalities and discrepancies between the findings of the studies were identified [16]. To perform this step, two reviewers read and re-read the data extracted for each study, noting patterns within and across the data. Tentative themes were then discussed within the broader research team over a series of formative meetings to reach consensus on the most pertinent themes arising from the data.

In the case of the literature reviews, only the data from relevant studies were included, and each qualitative study included within the systematic reviews was screened for inclusion as a primary study. Where studies included other groups of first responders in addition to paramedics, ambulance officers, ambulance volunteers, or call-takers, only the data relating to ambulance personnel was extracted and included. The quality of each included study was evaluated using the Critical Appraisal Skills Program (CASP) checklist for qualitative research, with quality of systematic literature reviews determined using the CASP tool of systematic reviews [17, 18]. The CASP checklists entail 10 questions related to various aspects of quality including whether aims were clearly stated, methodology, design, recruitment and sampling, ethical clearance, and rigor of data analysis. We determined high quality study were those that met eight or more of the 10 criteria, reasonable quality studies met 6–7 of the 10 criteria, and poorer quality studies met five or less of the 10 criteria. Where the reviewer assessing a study was unsure if the criteria had been met, input was sought from a second reviewer.

## Results

**Fig. 1:**
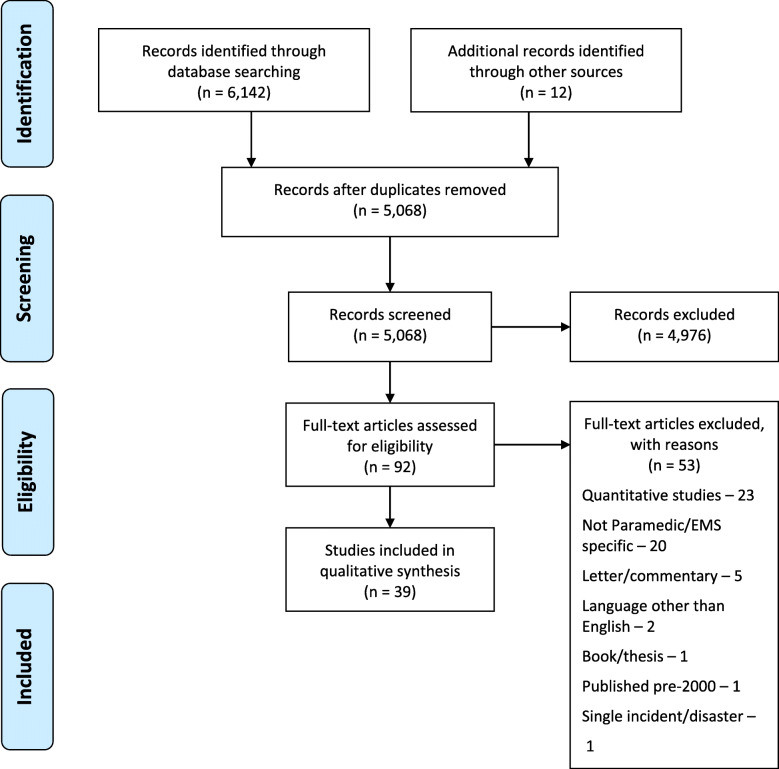
PRISMA 2009 Flow Diagram

A total of 6154 documents were retrieved from all searches, from which 1086 duplicates were removed. The remaining 5068 titles/abstracts were screened based on the agreed inclusion and exclusion criteria, with 4976 titles/abstracts removed because they did not meet the eligibility criteria. The remaining 92 full texts were reviewed, with 39 articles deemed to meet the eligibility criteria (see Table 2 and Fig. 1).

**Table 2:** Results retrieved from database searches

DATABASE	NUMBER OF RESULTS RETRIEVED
Ovid Medline (1946 to present), Epub Ahead of Print, In-Process & Other Non-Indexed Citations, and Ovid MEDLINE Daily	1604
CINAHL (1891 to present)	656
Ovid Emcare (1995 to present)	1260
PsycInfo (1806 to present)	2352
Scopus (1788 to present)	270
Google advanced search	12
Total before duplicates removed	6154
Total after duplicates removed	5068

The studies were conducted in a range of countries including the United States of America (*n* = 9) [9, 19–26], Australia (*n* = 8) [10, 27–33], Canada (*n* = 6) [34–39], Sweden (*n* = 5) [40–44], the England (*n* = 4) [44–48], Ireland (*n* = 2) [49, 50], Norway (n = 2) [11, 51], Saudi Arabia (*n* = 1) [52], and Israel (n = 1) [53]. Another study was conducted in both Australia and the United Kingdom [54]. Of the 39 articles, the majority were deemed to be primary research articles (*n* = 26) [20, 21, 25–30, 34, 35, 37–42, 44–46, 48–50, 52–54] with the remainder being either non-systematic literature reviews (n = 9) [9, 19, 20, 22–24, 31, 32, 36, 43] or systematic literature reviews (*n* = 4) [11, 43, 47, 51]. Of the primary research studies, the most common data collection method was semi-structured interviews (*n* = 11) [26, 27, 40–42, 44–46, 49, 51, 53]. Four studies used a combination of semi-structured interviews and focus groups [21, 34, 35, 54], while another four utilised questionnaires with at least one open-ended question [10, 20, 29, 37]. Six studies used a mixed methods approach, combining qualitative and quantitative approaches. These methods included a questionnaire and interviews (n = 4) [28, 30, 38, 39], conducting both interviews and focus groups, attending organisational management meetings and examining information available on the public record (*n* = 1) [48], and brainstorming, flow chart analysis, and examination of health service usage records (n = 1) [52]. Additionally, one study reported on the outcome of a court case pertaining to a paramedic who developed PTS as a result of his employment [25].

Of the 39 included studies, no studies focused explicitly on unpaid volunteers within ambulance services. Their views may have been captures within individual study samples; however, their status as volunteers was not distinguished from other participants. Hence, their views and experiences in relation to the review questions remains unknown. Only one study focused explicitly on retirement. This study from Ireland explored policies and procedures for retirement within ambulance and fire services, with a primary focus on managers aged 42–73 years [49]. One US study explored how EMS work impacts upon family life, with a convenience sample of 11 spouses and one parent of EMS providers [26]. The remaining 37 included studies related to ambulance personnel on active duty.

### Summary of quality evaluation

The majority of articles were generally of high quality, with all but one clearly stating the research aim and all using an appropriate research design. However, a qualitative methodology was not considered appropriate for one of the studies, and the appropriateness could not be determined for a further two studies. Recruitment strategies were deemed appropriate, except for three studies where insufficient details were reported. We were unable to determine if one study collected data in a way that addressed the research question; however, the remainder of the studies were deemed to have achieved this. Rigour of data analysis was considered acceptable in most cases except for one study, and five for which this could not be determined. Eight studies did not report on ethical issues and one did not provide sufficient information to determine this. The relationship between the researcher and the participants was adequately described in 12 studies; however, there was not enough detail to determine this in three studies. All studies concluded with a clear statement of findings (see Appendix 5). The four systematic literature reviews were deemed to be of high quality, with all stating a clearly focused question, and all considered to have adequately assessed the quality of included studies (see Appendix 6).

The results are summarised in two main sections, with each section addressing the specific research questions identified for this review:

1. Impacts and work-related psychological and physical well-being needs (Questions 1, 2 and 3)
2. Impacts or organisational and systems parameters (Questions 4 and 5)

### Section 1: impacts and work-related psychological and physical well-being needs

This section describes the psychological and physical effects of frontline emergency medical work on ambulance personnel. It addresses factors recognised by individuals and organisational management as being significant and contributing to mental health and well-being, or increasing the risk for developing conditions such as PTSD, depression, and anxiety. It also describes how work-related psychological and physical well-being needs were articulated and framed by ambulance personnel. Findings of the studies discussed in Section 1 are summarised in Appendix 3.

#### Q1: impacts on psychological well-being

Psychological effects were framed within five themes: 1) Recognised reactions and associated signs and symptoms; 2) Effects on relationships with others; 3) Observations from “above” (e.g., the influence of organisational structures, policy, and support); 4) Perceived control versus real control and the nature of the event; and, 5) Protective and coping mechanisms employed.

1. *Recognised reactions, associated signs and symptoms, and causes*

A number of key commonly-recognised responses to trauma and the associated signs and symptoms of adverse impacts of that trauma on ambulance personnels’ psychological well-being were identified. These responses included angry outbursts and changes in tolerance to everyday interactions, sleep disturbances and deficits, irritability, decreased social life, and an increased sense of isolation. An increase in vigilance and fear of doing the required role, and recognition of associated triggers, unwanted and unpredictable flashbacks or intrusive thoughts, fatigue, stress, high rates of sickness and days absent, and difficulty switching off were significant [9, 11, 24, 25, 27, 31, 34, 36, 39, 46, 50]. The high and increasing rates of depression and anxiety, and associated development of PTS and suicidal ideation [9, 11, 19, 20, 22, 23, 25, 34, 40, 41, 47, 52] were clearly outlined as current and systemic issues faced by paramedics and call-takers, with rates estimated as being twice as high as other health professionals [24, 25, 28, 30, 36, 39, 46, 47].

The nature of the work, including routine ‘everyday’ call-outs, and those that generated associated secondary or vicarious trauma, had a cumulative effect on ambulance personnel. This left them with feelings of frustration, helplessness, trepidation, and emotions of being overwhelmed during and after the event. The cumulative build-up of emotions and continued exposure to stress led to compassion fatigue and self-blame [39, 41, 45, 53]. The literature notes rates of substance use (e.g., alcohol and other drugs) by ambulance personnel accelerated following exposure to critical incidents as a means to mitigate and manage these lived experiences [9, 24, 34, 39].

One paper specifically outlined how attending critical incidents or traumatic events has six distinct stages related to the experience [42]. The initial stage involves anticipation of the event characterised as the (1) pre-trauma and preparation for the unknown. Early in the management of the event, there is a keen sense of (2) feelings of responsibility and the associated anxiety and fear of mistakes. As the event progresses, paramedics recognise intense feelings of being (3) insufficient and worthless, even though they have done everything possible accompanied by the challenges of (4) containing emotions and putting on the ‘professional face’. After the event, feelings of (5) confusion, exhaustion, and of being in chaos were dominant. Other more subtle feelings included being (6) rejected by relatives or fellow workers, feelings of anger, frustration, resentment, and bitterness, betrayal and rejection, self-loathing, guilt and humiliation, being out of control, trapped, and feelings of helplessness [42].

2) *Effects of relationships with others*

The effects of workplace stress on the psychosocial well-being of ambulance personnel profoundly influenced how they interacted with others. Social withdrawal and the above-mentioned signs and symptoms created an environment which negatively affected personal relationships, exacerbated the sense of isolation and withdrawal, and what they described as a debilitating loss of compassion. To manage this, paramedics specifically found themselves projecting difficult feelings and blame onto others, especially those close to them. This enabled them to distance themselves from their negative emotions and alleviate their emotional distress. Paramedics also found themselves being hyper-alert and over-protective of family and friends because they knew and saw worst-case scenarios as part of their everyday work [26, 34, 50, 53].

The effects of shift work added to the psychological stress due to the continuous negotiation between their role and identity at work and their personal life. Shift work specifically reduced the time for recovery and quality individual and family time with limits on their social life. Rosters, over-time, and other work commitments such as training and professional development often affected social and family gatherings at significant times of the year, and operating in a “9 to 5 world” was viewed as being potentially problematic. Paramedics described shift work as having a negative effect on family roles and intimacy, and disrupting the structure and rhythm of home life with concerns about family safety and the risks of the job [26].

3) *Observations from “above”: Occupational oversight and the operational environment*

The occupational environment and nature of the work performed by ambulance personnel was well recognised as being a significant contributor to stress, with a range of impacts on their mental and physical health. Prolonged and high exposure to excessive occupational demands and lack of crisis support led to poor physical and mental health, increased sick leave, and lower productivity [19, 26, 41, 50, 51], and was associated with increased overall morbidity, and physical and mental illnesses [19, 30]. Along with a challenging occupational environment, operational factors (such as key performance indicators, quotas, operational standards, response times, and expectations) also contributed to psychological stress [32, 45]. Another significant aspect was the feeling of a ‘big brother’ environment in which everything is observed and taped leading to call-takers being ‘on-edge’ [27]. The increased risk of potential aggression and violence that paramedics faced, particularly verbal abuse, added to the sense of work-related uncertainty and vulnerability. This was compounded if reports were not taken seriously or were under-reported, as culturally, potential agression and violence was seen as a ‘normal part of the job’ [28].

4) *Perceived control, real control, and nature of event/trauma*

The nature of the critical incident, and the perceived or real sense of control were identified as factors that either mediated or increased the incidence of psychological injury. Key events and case types that contributed to signficant distress for ambulance personnel included the death of a baby or child, neglect, abuse or harm, burns, assault, family violence, drownings, harm to colleagues [20], suicide, grotesque mutilation, and those cases that had a personal significance ascribed to them (e.g., personally knowing the patient, working with children or the critically ill, and the death of patients) [29, 38, 39, 43, 44, 50, 51]. Adding to the burden was the need to complete administrative and documentation requirements and to remain professional in further interactions with others following a critical incident.

Lack of control, whether perceived or real, particularly a lack of control over work, their environment, and work demands [28, 40, 47], increased the incidence and severity of psychological distress, and were triggers for the development, or exacerbation, of existing mental illness. Associated with the lack of control was the immediate knowledge of outcomes during the critical incident, or lack of clinical feedback after the event, which influenced how the critical incident was perceived and processed by ambulance personnel [24, 39, 47]. Other prominent issues in relation to feelings of loss of control and safety were workplace bullying, actual assaults by others (patients, colleagues), loss of control through verbal and physical threats, remoteness and isolation felt if subjected to these behaviours, lack of back-up support and reports of incidents not being taken seriously or not being followed-up by supervisors or the service.

5) *Protective and coping mechanisms*

Ambulance personnel used a number of strategies to cope with the nature of the work they face; some that contributed to the delayed nature of mental illness presentations. One strategy used was compartmentalising the event and associated emotions to manage the immediate demands and to be able to provide care which was protective in the short-term, but which may be detrimental in the longer-term, or they distanced themselves emotionally from the patient to protect themselves. Avoidant strategies and information searching were often used to try to regain a sense of control and manage the demands they faced [39, 45, 48].

The nature of the work, although complex and challenging for mental health, also offered individuals a sense of identity and status, affiliation and camaraderie, structure and routine, direction and meaning, and intellectual stimulation and challenge which together enhanced personal satisfaction and resilience. Social support, mainly emotional support, was also crucial and protective against the development of PTSD [51]. These protective aspects of the work are significant, but were often minimised or disrupted if a critical incident challenged their sense of role and connection to the service, especially if re-tasked or forced to medically retire due to mental health concerns or illness.

#### Q2: impacts on physical well-being

Physical side-effects of continued exposure to occupational stress manifested in predominantly somatic symptoms such as headaches, gastrointestinal distress, sleep disruption, fatigue, and their associated effects on work performance [9, 11, 24, 34]. The fast-paced nature of the work and the psychological demands of the job did not allow for physical rest and processing of incidents [38, 44], which created a vicious cycle for ambulance personnel, and contributed to a difficult work-life balance and poor post-shift recovery [27, 30, 36, 46].

Due to the nature of the work, the major physical concerns experienced by paramedics were reported as musculoskeletal injuries, specifically back problems, associated with the weight of patients and manual handling requirements in challenging environments. Other risks to physical health were blood-borne pathogens and needle-stick injuries which occurred mostly with inexperienced clinicians [22, 30, 44]. Critical incident stress particularly affected physical health due to associated weight gain, back problems, and changes in appetite and diet. It was difficult for paramedics to maintain or improve their general levels of fitness and maintain diet because of shift work and the lack of on-site exercise facilities [30, 50].

Sleep and disrupted sleep patterns are of interest as a key to understanding physical fatigue and psychological effects of work in this area. Inadequate or disrupted sleep has been associated with cardiovascular disease (CVD), metabolic disease, depression, impairment in immune function, and hormone secretion fluctuations which can instigate adverse psychological changes [33].

Both increases and decreases in cortisol levels have been found following stress exposure and indicate strain on the endocrine system [33]. The literature indicates that pro-inflammatory cytokines significantly increased or decreased from baseline following single as well as multiple nights of complete and partial sleep restriction [33]. Elevated levels of sleep regulating cytokines have been associated CVD, metabolic syndrome and depression; higher, flatter diurnal cortisol patterns are related to depression and elevated morning cortisol levels are positively associated with CVD and metabolic syndrome [33].

#### Q3: articulation of well-being needs

Ambulance personnel articulated their well-being needs in terms of occupational safety and in relation to their lived experience, across four key areas: organisational support; informal support; use of humour; and individual mechanisms to cope such as detachment and external supports [26, 43, 44, 47].

1. *Organisational and informal support:*

Ambulance personnel reported a need for broader recognition of incidents that may be viewed as routine and yet have personal significance, or were cumulative in nature and caused significant distress, thus becoming a critical incident [20]. To adequately address critical incidents, the literature highlighted the needs for structured recovery time after the event, active moves to address stigma surrounding mental health at all levels, ease of access to care, and supported case review in a non-judgemental environment with the focus on feedback and learning.

Both paramedics and call-takers identified a need for quality supervision, positive working relationships with managers and colleagues, and for workplace conflict to be taken seriously and addressed through education and training [34, 47]. Call-takers, in particular, identified that workplace conflict existed when their roles were not acknowledged. On occasion, they felt that paramedics did not value them and that they were seen as *‘punching bags’* in an *‘us and them culture’* [27, 46].

Paramedics particularly identified that workplace violence and threats were a significant and common issue and played a major role in their feelings of vulnerability and poor mental well-being. These issues need to be taken seriously by the organisation, with continued prevention and occupational safety measures implemented (e.g. duress alarms, recognition of dangerous addresses, increasing training and knowledge of staff of minimisation strategies and how to deal with violent patients) [20, 28].

2) *Individual mechanisms to cope*

Ambulance personnel described experiencing emotional pain arising from the work they do, with associated feelings of helplessness during and after extreme events. They described feelings of emotional and cognitive detachment from patients and their families during more routine events. They coped by focusing on the technical and clinical aspects of the job and the immediacy of the activity. More broadly, they expressed a need to maintain a sense of purpose and frame their work as meaningful [53]. They saw themselves as advocates and accustomed to using problem-solving and emotion-focused efforts to manage situations. These skills were considered essential to protecting oneself, as feeling useful and managing people and incidents was a positive and protective aspect of the role and contributed to their sense of identity. They also used advocacy skills as a means of addressing workplace stress and in attempts to get better resourcing (e.g. more personnel, equipment, and recognition) from management and the organisation to be able to perform their role effectively [43].

Ambulance personnel and their significant others employed cognitive strategies to manage not only the effects of their work environment, but also their relationships and life outside of work. These strategies included trying to go with the flow and realising that you can only control what you can, and to consider and allow yourself to look at the worst possible scenario as a means of cognitively and emotionally preparing for the demands of the role. During and after the event, it was essential to recognise early signs of distress and to know when to seek social support and how to cultivate those friendships and networks of support as essential preventative and positive means to reduce isolation. Negotiating family role responsibilities and learning how to balance relationships were identified as being crucial for personal well-being. As part of the balance of work and life outside of work, establishing and developing one’s own interests created a sense of meaning and assisted in delineating work and home life [26].

If illness, either psychological or physical or both, became evident, it forced a change in personal views of the self and highlighted the concept of striking a balance with the experience of wellness and illness. The sub-themes to this experience focused on the idea of attaining and maintaining wellness through personal nurturing, encountering illness as an experience and a threat, and accepting and managing illness. Wellness was nurtured by the experiences of being excited and challenged by the work they did or could still do; having freedom and flexibility and a sense of autonomy in their work and life; being “someone” and having a sense of making a difference; and being one of the gang [44]. If injury was severe, the positive and protective nature of being a contributing member was challenged and often created a sense of frustration, anger, and worthlessness. As part of framing wellness and illness, there was a distinct narrative of encountering illness as an experience and a threat. The illness experience had a psychological, physical and social dimension which was encapsulated by the concepts that “the body makes itself heard; one can get worn out; and one can become too vulnerable or hardened” [44].

### Section 2 impacts of organisational and systems parameters

The findings of the studies discussed in Section 2 are summarised in Appendix 4.

#### Q4: effects of workflow and nature of the work on mental health and well-being

The nature of the work performed by all first responders is distinguished from other occupations by exposure to human distress and tragedy, referred to in the literature as critical incidents [11, 20, 31, 35]. However, ambulance personnel share with all other professions and occupations issues to do with how work is organised, the industrial relations and human resources factors that have an impact on the job, the skills and qualities of managers who organise the work, and the personalities and experience of their immediate supervisors. Several studies note problems under this category of workflow [9–11, 19–21, 23, 27, 31–34, 37, 38, 40, 44–48, 50, 54].

1. *The type of work performed by ambulance personnel*

Ambulance personnel made a distinction between specific traumatic events that produce an emotional toll, such as attending the death of a child or a suicide of a young person [9, 20, 27, 41], and a patient who may present a range of problematic or difficult responses such as someone with a mental health issue [9], or domestic violence, a drug and alcohol incident, or road rage [37]. A further distinction was made under the broad heading of uncertainty or lack of control [38, 40, 47, 48, 55], for situations in which they must proceed to a job without adequate knowledge, unsure of what they will find or how to prepare themselves [37], or where they may be exposed to pathogens and it is difficult to ensure universal precautions [20, 27, 40, 47]. Any incident may therefore prove to be critical. The event does not need to be traumatic; it merely needs to evoke a strong emotional response in the individual [11, 41]. It may have an immediate impact on their sense of well-being, or in some cases, many months or years later, even beyond retirement, or precipitate early retirement [11, 49]. Small or major events had a cumulative effect on stress levels [11, 27, 31, 34], particularly if there was little time between jobs and shifts to switch off from the trauma of a previous incident [48, 54].

2) *The way the work is organised*

The organisation of work can be examined from the perspective of the strength of the industrial protections in place, and the expertise of the human relations and managerial systems within the organisation [38]. The research is almost universal in reporting that both are problematic for ambulance personnel. In dealing with industrial relationships arrangements, they report that given the workload pressures and performance metrics governing their jobs, they had no time to deal with, or digest, the effects of critical events [23, 27, 29, 33, 34, 45–47]. Other factors noted were lack of resources including a lack of available ambulances to send to a job [37, 38, 45], too few staff [10], and in the case of the USA, low salary requiring a second job [9], long shifts, lack of sleep linked to shift work, fatigue as a result of long drives in rural areas, and failure to have the required breaks or meals as a result of work intensification resulting in poor eating habits and weight gain [10, 20, 29, 31–34, 48, 54]. Added to this were the unreasonable metrics governing the speed required to get to a job, the time allowed ‘on-scene’ and later at the hospital before management radio in expecting the paramedic team to respond to the next case [23, 33, 45, 46, 50], and the erosion of team work and skills mix through the appointment of single responders, or lower grades of workers who lack the necessary skills [37, 45, 46]. For call-takers, stressful issues included working alone during weekends and at night, the intensity of the work that made it difficult to take breaks, and the antagonism of paramedics towards them [46, 50].

The general view reported is one of alienation between management and supervisors and on-road paramedics, ambulance officers, and volunteers [9, 34, 35, 48, 50, 54]. Managers were seen to lack empathy or consideration towards paramedic work, to downplay the impact of critical incidents, or the pace of the work [33, 46, 53], to stigmatise those seeking help [34, 35, 39], and to lack the skills and training to deal with workplace bullying [34, 35, 54]. Ambulance personnel reported not being supported in legal and audit cases and to consistently have their skill-base ignored through either a failure to be promoted, to access higher level roles, or unable to make their own clinical decisions [38, 48, 55]. A further frustration was the constant restructuring of the organisation [44].

#### Q5: effects of organisational structures on psychological and physical well-being

The organisational response to ambulance personnel’s psychological and physical well-being was influenced by the structure and culture of the organisation [38, 43] along with the personal attributes of the paramedic such as their age, gender, years of experience, and psychological factors such as internal locus of control and the capacity to ensure a work-life balance [9, 20]. In Australia, historically, these services had their origins in paramilitary culture, with a strong hierarchical chain of command, which in-turn prizes stoicism in the face of adversity, and compliance, with little sense of worker control or clinical autonomy [48, 54], but also a high level of teamwork, camaraderie, and public service [20, 38]. Paramedicine has moved from being a vocationally-trained, male-dominated, blue-collar occupation to a university-trained and registered profession with an increasing number of women and younger recruits who appear to be more comfortable with contemporary methods of stress management [34, 35]. Providing a comprehensive duty-of-care service to ambulance personnel along with meeting performance metrics during a time of cultural transition can prove difficult for any organisation [27, 46, 49].

1. *Managerial response to paramedic well-being and workplace stressors*

Managerial responses can be examined through the way managers respond to on-road or call-taker distress and to the type of welfare services provided [27, 34, 35, 45, 46, 48, 50, 54]. A number of studies noted that on-road staff report that managers universally failed to understand, appreciate, or respond to the distress of critical incidents [27, 30, 38, 41, 45, 48, 54]. The alienation of call-takers is particularly marked [46]. They reported that the only contact they had with management was when something went wrong, or the organisation wished them to implement an unpopular directive, leaving them to negotiate the ire of on-road staff [27, 46, 50]. Importantly, management’s lack of capacity to deal with organisational stressors such as bullying, workplace conflict, management of rosters and sleep deprivation, and promotion was also seen as a stressor that, in turn, exacerbated ambulance personnel’s reactions to traumatic incidents [9, 20, 29, 30, 38, 50].

Managers could assist through more thoughtful organisation of rosters to reduce fatigue and lack of sleep, along with ensuring adequate staffing to fill rosters and reduce overtime, and ensuring that rosters followed a clock-wise rotation [29, 31]. Managers benefitted from doing occasional shifts to keep themselves abreast of on-road circumstances [46], and for the need for adequate equipment [21]. Including call-takers in debriefing sessions along with paramedics was also seen as a positive strategy for managers to employ [27].

2) *Types of welfare services provided*

The type of welfare services provided to ambulance personnel, including those who have retired, and the evidence-base, is also complex, with reported ambiguity around the usefulness or otherwise of critical incident debriefing or mandatory crisis meetings [20, 23, 35, 41, 49], as well as cognitive behavioural therapy [22, 34, 47]. There was also debate about the time when management should offer services and what exactly should be provided [22, 41, 52]. Prevailing strategies promoted were pre-employment testing, peer support programs, and psychological first aid along with adequate training of front-line staff as well as managers and team leaders in order to recognise symptoms in individuals at risk [19, 34, 35, 46, 51]. In severe cases of PTSD, there was a recognition that pharmaceutical responses may be required until the individual gained emotional control [51]. There is limited research on pre-employment testing [32, 51]. Peer support can be as simple as time out between jobs, but time scarcity is a factor [34, 35, 44], and for younger personnel, time to learn to trust colleagues [34, 50]. One of the most important principles reported was to ensure confidentiality of counselling services [34, 35, 52] given the highly competitive nature of the contemporary workplace [35, 54], and that counselling staff be formally trained and experienced in understanding the issues faced by ambulance personnel [22, 35, 36]. Stress surrounding access to workers compensation was explored in the US context [25].

Training in stress management for managers was seen as essential, and for on-road staff was part of their personal armoury strengthening internal locus of control [9, 20]. The research suggested the cumulative impact of critical incidents made years of experience a negative factor in well-being, but also notes that experience was a positive factor [9, 20, 34, 48, 54]. While the individual was seen as primarily being responsible for maintaining their occupational well-being, the peer-reviewed literature also points to the responsibility of the employer to provide both welfare services and a working environment conducive to health [9, 20, 22, 38, 50].

## Discussion

This paper has provided a comprehensive overview of the qualitative peer-reviewed literature dealing with the impact of emergency medical service work on the psychological, physical, and social well-being of ambulance personnel. In synthesizing the literature, three key themes emerged which align with the three key areas of enquiry: What is the problem? What causes the problem? What is needed to address the problem?

### Theme 1 - mental health issues result from exposure to traumatic events, but also to the way personnel in the workplace respond (managers, peers) to worker distress

The peer-reviewed literature reported on the negative impact of continuous and cumulative exposure to critical incidents. There was strong agreement on the types of incidents that paramedics find particularly distressing; for example, those reported by Donnelly and Bennett [20]. There was also evidence to suggest that, on occasion, mundane jobs that may appear routine for one paramedic can trigger anxiety or distress for another, making exposure to stress an individual response. Recovery is made difficult by the nature of shift-work, over-time, the physical demands of the job, and fatigue. The way the work is organised matters and needs to be addressed with physical and emotional well-being in mind.

Both the Australian and international literature confirms that the incidence of psychological distress in ambulance personnel is not just a matter of exposure to traumatic incidents, but also arises from the way the organisation responds, at the managerial and organisational level. For example, at the managerial level, when paramedics experience work-related stress and require access to formal avenues of care, many managers are unsympathetic and lack personal empathy. This is often assumed to have its origins in the military-like or macho culture that discourages emotional displays of distress [48, 54].

Further to the above contributing factors, in several studies ambulance personnel reported that their access to appropriate care is made difficult through failure to acknowledge the stress, lack of confidentiality, use of inappropriate therapies, poor return-to-work mechanisms, isolation, and stigmatisation, concerted efforts to obstruct access to Worker’s Compensation provisions, and the lack of support post-retirement.

**Fig. 2:**
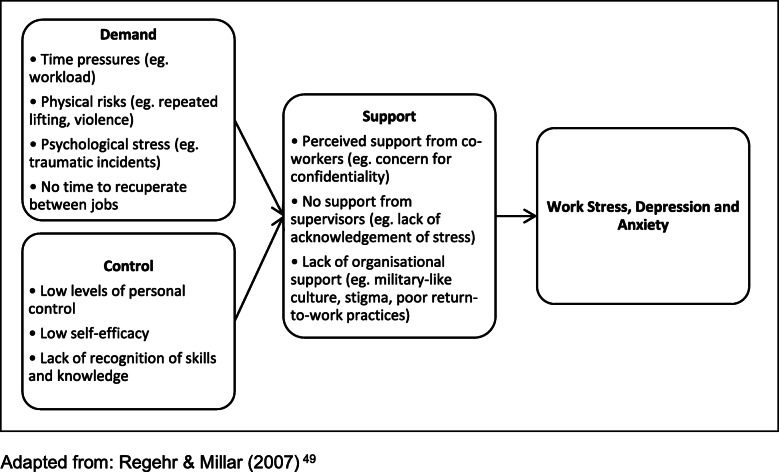
Conceptual model of paramedics’ work-related stress

The interactional impact between exposure to traumatic events and workplace culture goes beyond the lack of support or open hostility shown by some managers within paramedic organisations, to the negative outcomes associated with New Public Management principles. This is best summed up by Hamling, in relation to the New Zealand experience [55, 56], who argued that the productivity and efficiency targets set by governments and, of necessity, implemented by management, prevent on-road paramedics from achieving their *vocation to care*. She suggested that the culture of metrics (key performance indicators used by managers) means that on-road staff become more concerned about the speed in which a job is performed, than caring for the patient. This creates tension and stress in on-road paramedics and underpins their response to critical incidents and much of the workplace stress they experience. This view is supported by a number of other researchers in South Australia, the UK, and the USA, and was evident across the literature [9, 34, 35, 48, 50, 54, 56, 57]. Regehr and Millar encapsulate these issues within a framework of Demand and Control as factors affecting the individual’s sense of support. Where these factors are perceived to be or are absent, it leads to work stress and depression [38] (see Fig. 2).

### Theme 2 – ambulance personnel suffering from work-related stress feel abandoned by peers, management, and service, during illness, in return-to-work, and post-retirement

The research reported overwhelmingly that ambulance personnel perceive that they receive little support from the organisation when they are suffering from workplace stress, burnout, anxiety, and/or PTSD. They view mental illness as highly stigmatised within ambulance organisations, making them loathe to seek professional help. They report that informal helpful debriefing occurs largely outside of the service, with family or friends, despite most organisations providing peer support programs. Relying on outside support is not always satisfactory given the possibility that family and friends may experience vicarious trauma [58]. Strategies such as black humour are used, but this is increasingly seen as politically inappropriate, with paramedics on edge as to where and how it can be exercised [59].

Given the recognition that stress is cumulative, it is surprising that support for well-being does not extend to those personnel who are forced to retire due to illness or at the end of their career. There was little research reporting on support programs for retired personnel, or recognition that they may be able to assist those currently in the service in need of care. As noted, despite many services providing support mechanisms, at all points in their career, the individual is seen as responsible, with management and the organisation being unable or unwilling to assist. Some research suggests that the failure of management to respond to paramedic stress does not arise from a lack of empathy, but a failure in appropriate skills in managing bullying, conflict, and confidentiality which ‘boils over’ into workplace management [9, 20, 29, 30, 38, 50]. The overwhelming alienation between management and front-line ambulance personnel is a key finding emerging from a significant number of papers across the peer-reviewed literature.

### Theme 3 - policy, programmes and interventions, and education need to occur at an individual, peer, organisational, and government level (Programmes and resources)

Research exploring how the issues highlighted above can be addressed takes a holistic approach moving from the individual through to the organisation. The organisation can assist through a number of strategies and processes that can be divided between cultural shifts and organisational re-design. Cultural action is required to remove the stigma associated with seeking help for work-related stress. This is a difficult task and is probably only achieved when actions speak louder than any rhetoric on the topic. Actions taken up by the organisation will in turn shift cultural perceptions and include recognising that workplace stress is cumulative and that it can manifest following routine incidents. This may mean that the service needs to organise staffing and rosters to allow paramedics with structured recovery time between jobs so that fatigue and emotional processes can be addressed. This will invariably have an impact on budgets along with the need for a more concerted focus on the training and education of leaders and managers in all aspects of their role, from designing rosters that safeguard well-being, to recognising colleagues in distress, to the management of workplace tensions.

A number of studies reported mixed results from the support programs currently used within Ambulance Services. For example, there was considerable discussion on the use of Critical Incident De-briefing with claims that it lacked an evidence base. There was support for providing robust peer-support programs and independent counselling services that extended to ambulance personnel’s families and to those who have retired. Although the current recommendation is for psychological first-aid support programs, there is currently only limited evidence establishing efficacy of both peer support programs and psychological first aid in the emergency service context.

A number of studies suggested pre-employment testing, although there was insufficient evidence to support the recommendation [21, 51]. There is also recognition that younger employees may respond to different approaches, particularly if the organisation uses the internship period to demonstrate its commitment to workplace well-being. Researchers noted that counselling staff needed to be highly trained in the area, and to have greater knowledge of the day-to-day work performed by ambulance personnel [22, 35, 36].

Related to the above, it is clear from the research literature that the problems require ongoing education about mental health and wellbeing across the continuum from initial training to post-retirement. However, there was a notable absence within the literature on preparing student paramedics and other ambulance personnel for the potential psychological stresses associated with the role.

In response to the increasing need to care for those providing emergency medical responses, a number of national and international initiatives have been proposed and implemented. For example, in 2018, the Paramedic Association of Canada in partnership with other key organisations launched a new standard for developing and maintaining a psychologically healthy and safe workplace [60]. The standard specifically aims to assist ambulance organisations to: raise awareness of stigma, self-stigma and harassment; systematically identify potential sources of stress and hazards to psychological wellbeing; and identify measures that can be implemented to address those hazards. Naylor et al. [61] suggests there needs to be a concerted effort to integrate mental health into new models of care for frontline service personnel as part of efforts to increase mental health literacy and to support education and training.

A range of broad steps for policy are suggested by the findings of this review. For example, policies may be required to regulate the role and skills of counsellors working in this space, to ensure that they understand the unique nature of ambulance work are specialised in addressing the needs of this group who are routinely exposed to critical incidents. Policies should also consider how ambulance services’ support for injured ambulance personnel is funded. For example, this could include policies that provide members and their families (employed and retired) with financial support to seek their own confidential psychological well-being counselling outside of the organisation, with no financial cap put on resource use. Also, given the cumulative nature of psychological and physical impacts on this population, Workers Compensation claims systems and processes should be streamlined and accepted for conditions that are often chronic in nature and which require long-term support.

### Limitations

This systematic review had several limitations. There were few studies about retirement issues, none devoted to volunteers, and none that examined potential gender differences. Most studies involved mixed samples; however, experiences and needs may vary across gender, role, education level, urban and rural location, availability of supports, experience and seniority level. Research on the potential differences between various groups, such as those on active duty versus call takers, is still limited, and many of the included studies did not distinguish between groups when reporting study findings. To help address this problem, we recently examined all the included research on issues specific to call-takers [62]. Exposure to critical incidents and toll of shift work and full-time roster rotations may vary also across different groups within the population of ambulance personnel.

Other limitations include exclusion of research published prior to 2000, the exclusion of non-English literature, and terms used in some countries that may have been overlooked in the search strategy. There are also limits associated with qualitative research which relies on self-report, often involving sample sizes and single organisations for recruitment, which may have led to recruitment bias and problems with generalising the findings to other contexts and other countries. Further limitations included the inclusion of all available studies regardless of their quality which may have affected the trustworthiness of conclusions drawn and validity of themes.

## Conclusion

The research literature suggests that strategies to address psychological wellbeing of ambulance personnel are of two kinds: specific programs to assist them to manage the distress that comes with attending to critical incidents; and secondly, programs that deal with organisational issues. There is, however, insufficient evidence for generalising specific beneficial strategies programs across ambulance service systems and countries, more broadly; that is, to know whether one country is doing better or worse than others, and why. Further comparative research is needed. Despite the limitations noted, the research literature reported in this review points overwhelmingly to an interactional effect between critical incidents and workplace culture and demands. This culture includes day-to-day managerial actions and responses, but also the impact of shift work, poorly managed rosters, and long hours of work with little time between for recovery. Coupled with work-flow issues are the negative consequences of New Public Management productivity and efficiency targets, now part of many ambulance services that require the job to be done within particular time limits. There is sufficient evidence within the research literature to suggest that these metrics are detrimental to the mental and physical health and well-being of ambulance personnel.

## Supplementary information

**Additional file 1: Appendix 1.**. Systematic literature review search strategies

**Additional file 2: Appendix 2.** Description of studies included in systematic literature review

**Additional file 3: Appendix 3.** Impacts and work-related psychological and physical well-being needs

**Additional file 4: Appendix 4.** Impacts of organisational and systems parameters

**Additional file 5: Appendix 5.** CASP quality ratings of primary research and non-systematic literature reviews

**Additional file 6: Appendix 6.** CASP quality ratings of systematic review

## Data Availability

Data sharing is not applicable to this article as no datasets were generated or analysed during the current study.

## Abbreviations

AEASA: Ambulance Employees Association of South Australia
CVD: Cardio-Vascular Disease
EDC: Emergency dispatch centre
EMS: Emergency medical service
EMD: Emergency medical dispatcher
EMT: Emergency medical technician
PTS: Post-traumatic stress
PTSD: Post-traumatic stress disorder
