# Supplementary material for: The effects of emergency medical service work on the psychological, physical, and social well-being of ambulance personnel: a systematic review of qualitative research
Source: BMC Psychiatry. 2020 Jul 3;20:348. doi: 10.1186/s12888-020-02752-4 (PMC7332532; doi:10.1186/s12888-020-02752-4)
Supplement: Supplementary file 1 — Additional file 1: Appendix 1.. Systematic literature review search strategies [file 12888_2020_2752_MOESM1_ESM.docx]

**Appendix 1. Systematic literature review search strategies**

**Ovid MEDLINE(R) and Epub Ahead of Print, In-Process & Other Non-Indexed Citations, Daily and Versions(R)**

| 1 | adaptation, psychological/ or emotional adjustment/ or "sense of coherence"/ |
| --- | --- |
| 2 | Stress, Psychological/ or "Quality of Life"/ |
| 3 | Mental Health/ |
| 4 | (((Psycholog* or psychosocial* or emotion*) adj3 (wellbeing or well-being or "well being" or resilience or health or stable or stabilit* or skill*)) or (physical adj3 (health or wellbeing or "well being" or well-being or fitness or health)) or mental health or stress).tw,kf. |
| 5 | or/1-4 |
| 6 | emergency responders/ or emergency medical technicians/ |
| 7 | "Transportation of Patients"/ or Patient Transfer/ |
| 8 | (paramedic* or ambulance* or "Emergency Medical Technician*" or "Patient Transfer*" or "transport* patient*" or "First Responder*" or Out-of-hospital*or Pre-hospital* or "Emergency Medical Services" or "emergency medical dispatch*" or "emergency dispatch centre*" or "community paramedic*" or "intensive care paramedic").tw,kf. |
| 9 | or/6-8 |
| 10 | 5 and 9 |
| 11 | (case reports or letter or editorial or commentaries or note or protocol).pt. |
| 12 | 10 not 11 |
| 13 | limit 12 to English language |

**Ovid EMcare**

| 1 | psychological well being/ or mental health/ |
| --- | --- |
| 2 | emotional adjustment/ |
| 3 | *stress/ |
| 4 | *job stress/ |
| 5 | (((Psycholog* or psychosocial* or emotion*) adj3 (wellbeing or well-being or "well being" or resilience or health or stable or stabilit* or skill*)) or (physical adj3 (health or wellbeing or "well being" or well-being or fitness or health)) or mental health or stress).tw,kw. |
| 6 | or/1-5 |
| 7 | rescue personnel/ or paramedical personnel/ |
| 8 | patient transport/ |
| 9 | (paramedic* or ambulance* or "Emergency Medical Technician*" or "Patient Transfer*" or "transport* patient*" or "First Responder*" or Out-of-hospital*or Pre-hospital* or "Emergency Medical Services" or "emergency medical dispatch*" or "emergency dispatch centre*" or "community paramedic*" or "intensive care paramedic").tw,kw. |
| 10 | or/7-9 |
| 11 | (case reports or letter or editorial or commentaries or note or protocol).pt. |
| 12 | 6 and 10 |

**PsycINFO**

| 1 | exp Well Being/ |
| --- | --- |
| 2 | exp Physical Health/ or exp Mental Health/ |
| 3 | exp Coping Behavior/ |
| 4 | *STRESS/ or *OCCUPATIONAL STRESS/ |
| 5 | exp *Posttraumatic Stress Disorder/ |
| 6 | (((Psycholog* or psychosocial* or emotion*) adj3 (wellbeing or well-being or "well being" or resilience or health or stable or stabilit* or skill*)) or (physical adj3 (health or wellbeing or "well being" or well-being or fitness or health)) or mental health or stress).ti,ab,id. |
| 7 | or/1-6 |
| 8 | *Emergency Services/ or *Crisis Intervention Services/ |
| 9 | exp First Responders/ |
| 10 | exp Client Transfer/ |
| 11 | (paramedic* or ambulance* or "Emergency Medical Technician*" or "Patient Transfer*" or "transport* patient*" or "First Responder*" or Out-of-hospital*or Pre-hospital* or "Emergency Medical Services" or "emergency medical dispatch*" or "emergency dispatch centre*" or "community paramedic*" or "intensive care paramedic").ti,ab,id. |
| 12 | or/8-11 |
| 13 | 7 and 12 |
| 14 | (letter or editorial or note or commentary or case report or protocol).mp. [mp=title, abstract, heading word, table of contents, key concepts, original title, tests & measures] |
| 15 | 13 not 14 |
| 16 | limit 15 to English language |

**CINAHL**

| S12 | S5 AND S10 |
| --- | --- |
| S11 | S5 AND S10 |
| S10 | S6 OR S7 OR S8 OR S9 |
| S9 | TI ( (paramedic* or ambulance* or "Emergency Medical Technician*" or "Patient Transfer*" or "transport* patient*" or "First Responder*" or Out-of-hospital*or Pre-hospital* or "Emergency Medical Services" or "emergency medical dispatch*" or "emergency dispatch centre*" or "community paramedic*" or "intensive care paramedic") ) OR AB ( (paramedic* or ambulance* or "Emergency Medical Technician*" or "Patient Transfer*" or "transport* patient*" or "First Responder*" or Out-of-hospital*or Pre-hospital* or "Emergency Medical Services" or "emergency medical dispatch*" or "emergency dispatch centre*" or "community paramedic*" or "intensive care paramedic") ) |
| S8 | TI first responders or firefighters or paramedics or police or emergency services |
| S7 | (MH "Emergency Medical Technicians") |
| S6 | (MH "Emergency Medical Technician Attitudes") |
| S5 | S1 OR S2 OR S3 OR S4 |
| S4 | TI ( (((Psycholog* or psychosocial* or emotion*) N2 (wellbeing or well-being or "well being" or resilience or health or stable or stabilit* or skill*)) or (physical N2 (health or wellbeing or "well being" or well-being or fitness or health)) or mental health or stress) ) OR AB ( (((Psycholog* or psychosocial* or emotion*) N2 (wellbeing or well-being or "well being" or resilience or health or stable or stabilit* or skill*)) or (physical N2 (health or wellbeing or "well being" or well-being or fitness or health)) or mental health or stress) ) |
| S3 | (MH "Support, Psychosocial") OR (MH "Psychosocial Adaptation (Iowa NOC)") |
| S2 | (MH "Emotions") OR (MH "Psychosocial Adjustment: Life Change (Iowa NOC)") OR (MH "Emotional Support (Iowa NIC)") OR (MH "Caregiver Emotional Health (Iowa NOC)") |
| S1 | (MH "Adaptation, Psychological") OR (MH "Stress, Psychological") OR (MH "Psychology, Social") |

**Scopus**

TITLE-ABS-KEY (( psycholog* OR psychosocial* OR emotion* ) W/2 ( wellbeing OR well-being OR "well being" OR resilience OR health OR stable OR stabilit* OR skill* ) ) OR ( physical W/2 ( health OR wellbeing OR "well being" OR well-being OR fitness OR health ) ) OR mental AND health OR stress* ) ) AND TITLE-ABS-KEY ( paramedic* OR ambulance* OR "Emergency Medical Technician*" OR "Patient Transfer*" OR "transport* patient*" OR "First Responder*" OR out-of-hospital*or AND pre-hospital* OR "Emergency Medical Services" OR "emergency medical dispatch*" OR "emergency dispatch centre*" OR "community paramedic*" OR "intensive care paramedic" ) AND ( LIMIT-TO ( LANGUAGE , "English" ) )
