# Supplementary material for: The effects of emergency medical service work on the psychological, physical, and social well-being of ambulance personnel: a systematic review of qualitative research
Source: BMC Psychiatry. 2020 Jul 3;20:348. doi: 10.1186/s12888-020-02752-4 (PMC7332532; doi:10.1186/s12888-020-02752-4)
Supplement: Supplementary file 2 — Additional file 2: Appendix 2. Description of studies included in systematic literature review [file 12888_2020_2752_MOESM2_ESM.docx]

**Appendix 2: Description of studies included in systematic literature review**

| **Author, year, country** | **Aim & methodology** | **Sampling & participant/study characteristics** | **Data collection methods** | **Data analysis methods** | **Limitations** |
| --- | --- | --- | --- | --- | --- |
| Adams et al. [27]  2015  Australia | Explore the lived experience of emergency medical dispatchers (EMDs) to understand how best to promote mental health and well-being.  Interpretive phenomenology. | 35 volunteers responded to work email invitation, then random selection of those approached for interview. This was followed by strategic targeting to ensure a mix of age, gender, region, experience level.  N = 16, 6M/10F, 24-57 years old, 2-15 years’ experience. | Semi-structured in-depth interviews via phone or Skype. | Interpretive Phenomenological Analysis (IPA). | May not be generalisable to all EMD roles due to individual and group variability, cultural influences, and operational and organisational procedures. |
| Alzahrani et al. [52]  2017  Saudi Arabia | Investigate the reasons for low usage of psychological support services among emergency medical service (EMS) staff and implement interventions to increase this usage. Evaluation of an intervention. | A multi-disciplinary team investigated the causes of low service usage, including a team leader, psychiatrist, psychologist, station manager, information technologist, and general director. Contact was made via email.  Participant characteristics unknown. | For analysis of the causes of low usage, consultation with multi-disciplinary team involving brainstorming, fishbone diagram, and flow chart analysis. For evaluation of the interventions, psychological service usage records were tracked producing quantitative data. | For analysis of causes of low usage, root cause analysis was implemented. For evaluating the interventions, changes in psychological service usage were tracked during specified periods. | Minimal detail provided regarding participant characteristics. |
| Avraham et al. [53]  2014  Israel | Explore the lived experience of paramedics related to critical incidents and associated coping strategies.  Phenomenology. | Purposive sampling through direct contact with paramedics (specific medium of contact unspecified).  N *=* 15, 10M/5F, 23-51 years old, 1-26 years’ experience. | In-depth semi-structured interviews conducted out of work hours that were 1-3 hours in length. | Thematic content analysis. | Small purposive sample in one jurisdiction that limits generalisability to other countries. |
| Bledsoe & Barnes [19]  2003  USA | Provide a critical review of Critical Incident Stress debriefing. | Refers mainly to literature review by McNally et al. (2003). Number of studies included unclear. Types of study designs include meta-analyses, RCTs, quasi-experimental. | Unspecified. | Unspecified. | Relies mainly on findings of an existing literature review. |
| Bracken-Scally et al. [49]  2015  Ireland | Explore policies and procedures for retirement in the emergency services. | Purposive sampling across ambulance and fire services in both urban and rural areas, with a primary focus on managers.  N = 14, 13M/1F, 42-73 years old, average years’ experience = 27. | Semi-structured interviews with predefined topics via phone or face-to-face, 30 minutes in length. | Framework analysis. | Focused primarily on still-employed individuals despite a focus on retirement. |
| Chappell & Mayhew [28]  2009  Australia | Highlight the risks of violence to operational ambulance officers and to identify the consequences for their physical and mental well-being. | Recruitment approach not stated.  Sample size represents 1 in 66 ambulance officers in the Australian public health system. Sub-set of a larger study of 400 health workers.  N = 40, 29M/11F. | Standardised, semi-structured interview-based questionnaire (including quantitative and qualitative questions). Conducted primarily on a 1-1, face-to-face basis. | Qualitative data analysis methods not stated. | Minimal detail provided regarding study methodology. |
| Clompus & Albarran [45]  2016  England | Explore the question of how paramedics survive their work within the current healthcare climate specifically by focusing on what resilience strategies they have and how childhood experiences may have developed these resilience strategies. | Participants recruited through advert in paramedic bulletin.  Paramedics were from one UK service.  N = 7, 2M/5F, 30-59 years old. | Narrative interview free association, then semi-structured interview. | Thematic analysis. | 5 of the 7 participants were female. |
| Coxon et al. [46]  2016  England | Explore the lived experience of EMD staff to understand how best to identify the key stressors and their impact on staff well-being. | Purposive sampling from an emergency office with total staff of 36.  N = 9, 5M/4F, 26-60 years old, 2-14 years' experience. | Semi-structured, in-depth face-to-face interviews. | Interpretive study using Braun and Clark’s 6-step method. | May not be generalisable to all EMD roles due to individual and group variability, cultural influences, and operational and organisational procedures. Researchers had little experience or knowledge of the area. |
| Donnelly & Bennett [20]  2014  USA | Develop an instrument or inventory of critical incidents exposure and to test the correlation between critical incident exposure and PTS. | Purposive sampling with 12,000 EMT and paramedics contacted.  N = 1,633 responders, 1208M/418F, median years of experience = 6 years. | Online questionnaire including one qualitative question. | Statistical analysis of quantitative data and thematic analysis of qualitative data. | Only 13% responded from total population, and only one question on the questionnaire was qualitative. |
| Donnelly & Siebert [9]  2009  USA | Systematic literature review with primary focus on developing a model that demonstrates the relationship between PTSD and PTSS and alcohol and drugs. | Empirical articles using emergency medical responders. Additionally, given limited literature, other study designs (e.g., theoretical) and related populations also included (e.g., police). | Major search engines used: Medline, PsychInfo, Cambridge Scientific Abstracts, Articles First/WorldCat, and Google Scholar. | Not applicable. | Limited literature base to draw from. |
| Dropkin et al. [21]  2015  USA | Identify main work-related health problems among EMS workers in the United States; identify risk factors at the organisational, task, and exposure level; identify prevention strategies; examine these issues between participants (EMS workers and supervisors).  Grounded theory. | Convenience sampling from an EMS medical services unit in North Eastern USA.  N = 58, 58M/0F, mostly under 35 years old, slight majority of sample with greater than 8 years’ experience. | In-depth interviews with paramedics and focus groups with EMS team leaders. | Grounded theory approach to describe thematically what each group wanted followed by comparison between EMS workers and team leaders and managers. | All male sample. |
| Flannery [22]  2015  USA | Explore the rationale for introducing multi-modal treatment response to PTSD in first responders: police, firefighters, and paramedics. | Empirical articles focusing on trauma in first responders reviewed. Minimal information provided regarding study characteristics. | Unspecified. | Unspecified. | Limited literature base to draw from. |
| Forslund et al. [41]  2004  Sweden | Analyse the situations that emergency operators experienced as difficult to deal with and their reflections on how they managed them.  Phenomenological hermeneutic approach. | Purposive sampling of telephone emergency operators recruited from one centre via letter outlining research.  N = 16, 6M/10F, 34-56 years old, average years of experience = 15. | Individual interviews conducted at call centres. | Three stages: naïve reading, structural analysis, and interpreted whole. | Interviews should have been done elsewhere. |
| Gallagher & McGilloway [50]  2008  Ireland | Evaluate the impact of critical incidents on frontline staff by allowing them to tell their own stories. | Conducted in Health Board with population of 1.6 million and large service with radius of 622 square miles.  N = 27, 27M/0F, 31-60 years old, slight majority with more than 16 years’ experience. | Interviews based on a literature review and findings from stage one of research. | Thematic analysis, but methodological approach not noted. Analysis done by both authors, sharing in reading a random sample of transcripts. | All male sample. |
| Gist & Harris Taylor [23]  2008  USA | Outline the requirements of the organisation and the individual to maintain mental health and avoid PTSD. | Empirical articles focusing on how to maintain health of EMS staff. Minimal information provided regarding study characteristics. | Unspecified. | Unspecified. | Review methodology not documented. |
| Golding et al. [47]  2017  England | Investigate and synthesise available evidence relating to the psychological health of emergency dispatch centre (EDC) operatives and identify key stressors that they experience. | 2,358 articles retrieved, 16 accepted.  Included qualitative and quantitative studies.  Inclusion criteria:   - Participants were emergency (ambulance, fire, police) call-handlers and dispatchers (EDC operatives) working in EDCs - Any intervention, where applicable - Comparator is either another intervention, or no intervention, where applicable - Any psychological health outcome measures in relation to working within an EDC - Any study design | 8 databases were searched: Embase, PubMed, Medline, CINAHL, PsychInfo, PsychArticles, The Psychology and Behavioural Sciences Collection and Google Scholar.  PICO acronym used for inclusion. | Narrative description using CASP checklist. | Limited literature base to draw from. |
| Halpern et al. [34]  2009  Canada | Characterise critical incidents experienced by ambulance workers as well as elicit suggestions for interventions. | Sample taken from 900 paramedics and 100 supervisors attending an educational workshop.  N = 60, 33% female, mean age = 33 years, mean years of experience = 13. | Interviews and focus groups. | Ethnographic content analysis used.  Qualitative exploratory method used to understand critical incidents from the point of view of participants. | All participants were self-selected. |
| Halpern et al. [35]  2009  Canada | Explore and describe Emergency Medical Technicians’ (EMTs) experiences of critical incidents and views about potential interventions in order to facilitate development of interventions that take into account EMS culture. | An iterative sampling approach was used to ensure that both genders and all job levels were represented, and to identify when saturation had been reached.  N = 60 (4 supervisors, 56 EMTs), 33% female, average age = 39 years, average years of experience = 13. | Focus groups and individual semi-structured interviews. | Ethnographic content analysis and constant comparative method. | Although the EMTs who volunteered to participate in the study were representative of the EMS organisation in terms of age and years of experience, the participants may have higher levels of traumatisation, more vocal, or differ in other ways to the majority of EMTs. |
| Hegg-Deloye et al. [36]  2014  Canada | Identify the literature available on the effect of paramedics’ jobs on their health status. | Studies were included if the experimental protocol was conducted among professional workers and included a literature review. Experimental studies with fewer than six participants, and observational studies with a questionnaire response rate below 60% were not included.  25 studies were included in the review. | Electronic databases used: MEDLINE (Ovid, PubMed, National Library of Medicine) between 2000 and 2011. | Not applicable. | The shifts reflected on in some studies were not as demanding as shifts when major accidents occur. |
| Hugelius et al. [41]  2014  Sweden | Survey ambulance managers’ experiences of crisis support interventions for ambulance staff after potentially traumatic events. | Ambulance managers from ambulance stations including urban and rural areas were recruited via telephone.  N = 6, gender unspecific, 1-13 years of experience. | Semi-structured interviews using an interview guide developed based on the literature and the authors’ experience. | Content analysis. | The interviews were not recorded, transcription occurred via the 2 authors taking “literal notes” during the interviews. All participants were from 1 health care region. |
| Jonsson & Segesten [43]  2004  Sweden | Uncover and deepen the understanding of the way ambulance staff experience and handle traumatic events and to develop an understanding of the life world of the participants.  Descriptive phenomenology and an interpretative Heideggerian approach. | Participants were recruited strategically to ensure a variation in terms of age, educational background, and experience as ambulance staff.  N = 10, specifics of gender, age, and experience unspecified. | Interviews were audio-taped and transcribed verbatim. | Transcripts analysed using 5-step process involving: 1) reading whole interview, 2) search for meaning units, 3) synthesise meaning units, 4) organise embodiments, and 5) a general structure was verbalised. | Small sample size and minimal information regarding participants. |
| Klimley et al. [24]  2018  USA | Examine research regarding PTSD in police officers, firefighters, and emergency dispatchers with particular attention to the prevalence, comorbid diagnoses, risk and protective factors, and resources available to each group. | Eligibility criteria: 1) Sample included first responder group, 2) Study used a validated PTSD measure, 3) Participants had no indications of history of head trauma or serious psychiatric conditions.  218 studies were included. | Google Scholar and PsycInfo searched between 1960 and 2018. | Narrative review of themes including prevalence, comorbid diagnoses, risk and protective factors, and resources available to each group. | It may be difficult to recruit participants for research studies relating to PTSD, and therefore ascertain its prevalence, due to first responder culture and perceptions of potential occupational repercussions, skepticism and fears around confidentiality. |
| Larsson et al. [44]  2016  Sweden | Synthesise existing research on daily hassles in professional first responder settings into a theoretical model. | Eligibility criteria: 1) Peer-reviewed research paper, 2) Written in either English, German, Spanish, or Scandinavian languages.  40 studies were included. | Identical searches were conducted in the following databases: CINAHL, ERIC, Medline, PsycINFO and Sociological Abstracts (covering the whole time- period of each database). | The CASP tools for qualitative and quantitative studies were used to assess the quality of the studies.  Descriptive thematic analysis.  The data displays were coded and codes regarded as related were combined into categories. Following this, categories were compared and superior categories were developed. Codes, categories and superior categories were constantly being checked against the data displays and the original articles. | Only one of the included studies utilised a qualitative method.  Lack of intervention studies means suggestions cannot be more specific than what has been established in the general research on work and stress.  Only seven studies looked at more than one group of professional first responders. This does not allow for reliable conclusions regarding sub-group differences.  Despite the authors’ efforts, it is possible that not all the relevant studies were included in the review. |
| Lindahl [25]  2004  USA | Discuss the decision of the Virginia Supreme Court in a case in which a firefighter/ paramedic who developed chronic disabling PTSD after responding to a fatal fire was initially denied benefits as he had previously reported symptoms prior to the incident. | 44 year-old male firefighter/ paramedic with 19 years professional experience. | Not applicable. | Not applicable. | None identified by the author. |
| Mahony [48]  2005  UK | Explore the occupational stressors experienced by ambulance personnel following corporate restructuring.  Ethnography. | 28 ambulance personnel.  Recruitment approach not specified, however part of the sample was selected by the ambulance organisation.  To ensure representativeness, attempts were made to draw quotas of participants from strata by sex, geographic location, and rank. | Semi-structured face-to-face interviews, focus groups, attendance at organisational management meetings and examining information available on the public record. | All interviews were audio-recorded, transcribed, and analysed. | Part of the sample (9/28) was selected by Divisional Commanders who decided officers would be “articulate” and “ambassadors for the service”. |
| Mahony [54]  2001  Australia & UK | Explore the aetiology of occupational stress experienced by on-road ambulance officers. | Participants were approached in person and recruited to fill 3 quotas: rank, gender, and metropolitan/rural.  60 ambulance officers, paramedics, and patient transport officers. | Semi-structured face-to-face interviews using questions developed from previously conducted focus groups. | Interviews were tape-recorded, transcribed, and all comments coded. Recurring comments and themes were deemed to have more validity than the odd anecdotal story. Body language, pauses, reluctance to talk about a subject, and changing the subject were all noted, as was any emotive language or changes in volume. | The UK sample was less representative than the Australian sample as some of the UK sample was selected by Divisional Commanders who decided which officers would be “articulate” and “ambassadors for the service”.  UK personnel, apart from being told they were to act as ambassadors for the service in any dealings with outsiders, were cautious about saying anything that could jeopardise their jobs and incomes in a very limited job market. |
| Paterson et al. [10]  2014  Australia | To explore factors paramedics recognise as contributors to fatigue. | Convenience sample of paramedics.  N = 49, 24% female, mean age = 38 years, 12 (24%) female, 41% had been employed by an ambulance service for between 5–10 years. | Data were collected as part of the demographic questionnaire from a larger cross-sectional survey study. | General inductive approach. | Given the survey measure used, the ability to elicit detailed responses from paramedics was limited.  There is no current consensus on the definition, which might have influenced responses.  Participants were relatively small convenience sample, which might limit external validity of the findings. In particular, differences in understanding fatigue might exist between rural and urban paramedics, or between paramedics working in partnerships or teams, compared with paramedics operating alone. |
| Pow et al. [37]  2017  Canada | To examine whether the detrimental effects of daily occupational stress on sleep quality were buffered by perceived social support availability. | Paramedics self-selected into the study in response to online media as well as flyer and brochure advertisements posted at local EMS stations. The advertisements directed interested paramedics to an online website where they were asked to complete an eligibility questionnaire.  N = 87, 82% male, mean age = 42 years, average years of experience = 15 years. | Participants completed structured diaries for 7-day period along with a final online questionnaire battery. | Qualitative data (free text, open-ended survey question on workplace stressors) were summarised as the % of workdays each particular stressor was reported.  Quantitative data was analysed using hierarchical linear modelling (HLM) and multiple regression. | Sleep quality was self-reported with a single item.  Measuring occupational stress using an 11-item checklist may not capture perceptions of the severity of stressors experienced or the full range of stressors experienced during a typical workday.  Sample was relatively small and homogeneous. All participants were in cohabiting romantic relationships, with most being male and Caucasian. Thus, generalisability was limited to these groups.  Small number of females in the sample may have rendered the study underpowered to find gender effects. |
| Pyper & Paterson [29]  2016  Australia | Investigate levels of fatigue, stress, and emotional trauma in rural and regional ambulance personnel. | Convenience sample of rural and regional ambulance personnel. 577 contacted.  N = 134, 103M/31F, 21-60+ years old, average years of experience = 13. | Online questionnaire including both quantitative and qualitative elements. | Descriptive analysis/deductive content analysis. | Predominately male, paramedic population.  Limited representation across classification of paramedics.  Self-report measures were utilised. |
| Regehr & Millar. [38]  2007  Canada | Explore how paramedics perceive different facets of jobs including demands, degree of control, and level of support. | Convenience sample of urban paramedics.  Quantitative component of research: N = 86, gender unspecified, age range 26-56 years, average years of experience = 15.  Sub-sample of 17 used for qualitative interviews. | Questionnaires and interviews. | Demand/control/support model as a framework for analysis. | Gender of participants unspecified. |
| Regehr et al. [39]  2002  Canada | Explore the experiences of paramedics in a large urban emergency service organisation with regard to working with victims of violence. | Convenience sample of paramedics.  N = 86, gender unspecified, 26-56 years old, average years of experience = 15. | For quantitative component, a variety of measures looking at exposure to traumatic events, levels of social support, and levels of distress were used. Semi-structured interviews used for qualitative component. | Open coding broad themes developed and selective coding for meaningful narrative of the experience. | Sampling for the quantitative component/ qualitative data not intended to be generalisable and specific to group and organisation. |
| Rice et al. [30]  2014  Australia | Examine perceptions of current physical health status and the relationships between stress level and physical health and job satisfaction, in nurses, midwives, and paramedics in Australia. | Participants were recruited by advertisements in health care and professional organisations, using a snowball selection technique.  N = 24, 3M/21F, average age = 39, average years of practice = 16. | Semi-structured interviews and a self-rated stress survey questionnaire collected in 2012. | Thematic analysis and descriptive statistics. | Uneven distribution of health professionals (4 paramedics, 15 nurses, and 5 midwives). |
| Roth & Moore [26]  2009  USA | Explore how EMS work impacts upon family life.  Phenomenological approach. | Convenience sample of 12 participants (11 spouses and one parent of EMS providers).  Participants were recruited from three EMS systems, 2 suburban, and 1 urban. | Semi-structured qualitative interviews. | Thematic analysis. | Small convenience sample from 3 services, but appropriate for phenomenological approach. |
| Skogstad et al. [51]  2013  Norway | Review the research on occupational groups that are at particular risk of developing work-related PTSD. | First selection process:  Inclusion criteria: Published in the English and Scandinavian languages  Exclusion criteria: Articles on treatment and PTSD not relevant for occupational settings; studies of military personnel and ‘9/11’  360 studies were included.  Second selection process:  Inclusion criteria: peer-reviewed studies with highest scientific quality starting with longitudinal studies, systematic reviews, and cross-sectional studies with more than 100 participants. Clinical studies with more than 10 participants also included.  140 studies were included. | Literature search conducted in the following databases: OVID MEDLINE, OVID Embase, Ovid PsycINFO, ISI Web of Science, and CSA Health and Safety Science Abstracts. | Not applicable. | Most studies on work-related PTSD have been cross-sectional with very low response rates, and in many of the studies, workers responded to a questionnaire where they reported symptoms and exposure levels.  Risk of self-report bias in these studies, which could lead to an ‘artifactual covariance between the predictor and criterion variable’ due to the fact that the same person is assessing both measures. |
| Sofianopoulos et al. [31]  2012  Australia | Identify the literature available on pre-hospital providers regarding the effects of shift work on sleep. | Articles were included if they contained information relating to sleep disturbance/ disorder, fatigue, and work-related stress in the pre-hospital setting. Letters and editorials were excluded. | Electronic databases were: the Cochrane Database of Systematic Reviews, Ovid MEDLINE, Proquest, AMED, and CINAHL.  9 studies met the inclusion criteria with another 3 sourced from references in the retrieved papers. |  | More detail needed on the inclusion and exclusion criteria and the way the articles were confirmed in the analysis. |
| Sterud et al. [11]  2006  Norway | Systematically explore the literature on health problems and work-related and individual health predictors in the ambulance services. | Inclusion criteria:  A peer-reviewed original study, in which either health status separately, or the relationship between health and stressful working conditions or individual differences in ambulance services was assessed.  49 studies were included. | Electronic databases included: Medline, EMBASE, PsychINFO, CINAHL, and ISI Web of Science. Other relevant sources were identified through reference lists and other relevant studies known by the research group. | Studies ranged according to level of evidence based on sample bias, lack of comparison groups, and design. Studies that compared ambulance workers using the same methods and instruments were most adequate, and prospective studies were considered most adequate. | Small sample sizes, non-representative samples, and lack of comparisons with normative data limited the interpretation of many studies. |
| Varker et al. [32]  2018  Australia | Explore what current evidence exists regarding the mental health and well-being of Australian emergency services personnel. | Inclusion criteria:   - Australian emergency services personnel - Focus of the study was mental health or well-being - Study presented original research.   43 studies were included; a further 6 secondary studies. | Broad search of relevant databases was conducted: the PsycINFO, EMBASE, and the Cochrane Library databases were searched for relevant peer-reviewed literature 2011-2016 inclusively. | Not applicable. | No assessment of the quality of the studies, or bias in their methodologies was made for the current study, meaning that it is not possible to make judgements about the quality of research conducted within this area. |
| Wiitavaara et al. [40]  2007  Sweden | Explore the experience of illness and wellness in ambulance personnel with musculoskeletal symptoms.  Grounded theory. | Male ambulance personnel purposefully selected from an epidemiological study focused on musculoskeletal symptoms. | Narrative interviews. | Codes were grouped based on content and emerging ideas; key, intermediate, and sub-categories. | All participants were male and from one ambulance service. |
| Wolkow et al. [33]  2015  Australia | Critique the emergency service literature that has investigated the effects of sleep restriction on hormonal, inflammatory, and psychological responses.  Additionally, to investigate if a psycho-physiological approach can help contextualise the significance of such responses to assist emergency service agencies monitor the health of their personnel. | Inclusion criteria for studies:  Active duty emergency personnel / defense personnel or those in physically demanding positions which had similar sleep restriction patterns.  Complete or partial sleep restriction (i.e. < 7 h sleep) 1–8 consecutive nights.  Single day or consecutive shifts with periods of restricted sleep - no specific night shifts  Physiological stress responses pro- and/or anti-inflammatory cytokines and/or cortisol  English-language studies  published 1985- 2013, | Databases searched were: Allied and Complementary Medicine Database, CINAHL, Global Health, Health Source (Consumer and Nursing/ Academic Editions), MasterFILE, MEDLINE/ PubMed, PsycARTICLES, PsycBOOKS, PsycEXTRA, Psychology and Behavioral Sciences, PsycINFO, PsychTESTS and SPORTDiscus). | Not applicable. | Sleep and stress response research to date has focused mainly on soldiers and not other emergency responders or used the wider stress response literature which replicated similar sleep patterns to emergency service personnel to support discussion. |
