# Supplementary material for: The effects of emergency medical service work on the psychological, physical, and social well-being of ambulance personnel: a systematic review of qualitative research
Source: BMC Psychiatry. 2020 Jul 3;20:348. doi: 10.1186/s12888-020-02752-4 (PMC7332532; doi:10.1186/s12888-020-02752-4)
Supplement: Supplementary file 3 — Additional file 3: Appendix 3. Impacts and work-related psychological and physical well-being needs [file 12888_2020_2752_MOESM3_ESM.docx]

**Appendix 3: Impacts and work-related psychological and physical well-being needs**

| **Author, year, country** | **Question 1: Impacts on psychological well-being** | **Question 2: Impacts on physical well-being** | **Question 3: Articulation of well-being needs** |
| --- | --- | --- | --- |
| Adams et al. [27]  2015  Australia | Key impacts: decreased social life, hypervigilance, unwanted and unpredictable flashbacks and triggers as part of ongoing role, ‘big brother’ environment where everything is observed and taped leading to EMDs being ‘on-edge’. | Fatigue. | EMDs perceive that paramedics ‘see us as their punching bags’ with an ‘us and them’ culture.  On their days off, they do ‘something nice’, i.e., self-nurture, self-reward. |
| Alzahrani et al. [52]  2017  Saudi Arabia | Presumed to result in stress and anxiety and depression linked to the nature of the work. | Not discussed/noted or the focus of the paper. | Not discussed/noted or the focus of the paper. |
| Avraham et al. [53]  2014  Israel | Severe frustration and helplessness, terror, overwhelmed during and after the event, self-blame, projecting difficult feelings/blame onto others enabled paramedics to distance themselves from negative emotions, to alleviate emotional distress. | Not discussed/noted or the focus of the paper. | Most described focusing their thoughts on the technical activity and management procedures on the way to the event, then during the event they described experiencing emotional pain, compassion, and helplessness (in extreme situations) and emotional detachment (more routine events) and emotional, cognitive, and physical detachment from the patient’s family members. Then plagued by difficult emotions and thoughts after the event, feeling helpless, terrified, and emotional detachment to prepare for going home from work.  Overall, control and lack of control, compassion and detachment cycles as coping strategies. Reframed cognitively to see their work as meaningful and rewarding. |
| Bledsoe & Barnes [19]  2003  USA | May cause PTSD. | Not discussed/noted or the focus of the paper. | Not discussed/noted or the focus of the paper. |
| Bracken-Scally et al. [49]  2015  Ireland | Widely acknowledged that prolonged exposure to excessive occupational demands can lead to poor physical and mental health, increased sick leave, and lower productivity.  Occupations that involve a high level of stress report below average levels of physical health, psychological well-being, and job satisfaction.  Stress and chronic disease in ageing and retired workers found that prolonged stress was associated with overall morbidity and numerous physical and mental illnesses.  “Defined” by the occupation, and consequently, loss of occupation can lead to a profound sense of loss, boredom, and feelings of uselessness. Benefits of employment that are often missed by retirees include: (a) identity and status, (b) camaraderie and affiliation, (c) structure and routine, (d) direction and meaning, (e) personal satisfaction, and (f) intellectual stimulation and challenge. | Not discussed/noted or the focus of the paper. | Not discussed/noted or the focus of the paper. |
| Chappell & Mayhew [28]  2009  Australia | NOTE: Impact was examined using quantitative methods. The qualitative component of the study focused on the nature of the events experienced by ambulance officers.  Participants spoke of a variety of incident types. Most were perpetrated by patients; however, some were by other ambulance officers:  *Other ambulance officers:*   - Bullying from another ambulance officer - Time off work after being physically and sexually assaulted by another ambulance officer   *Patients/patients’ families/bystanders*:   - Verbally and/or physically threatened (including with weapons), having furniture thrown at them, being sexually assaulted, being bitten, being chased, being held captive, going to remote communities alone despite there being a high rate of violence   Particular cases pose a higher risk of violence than others (e.g. domestic violence incidents, mental health cases, certain geographical locations, drug and alcohol intoxication, young males). Verbal abuse seen as almost “normal” part of the job, but “never” followed up if ambulance officers report it. In most cases, no consequences for the perpetrator unless police were called or already at the scene.  Quantitative summary: Ambulance officers had higher levels of stress than the general population; those from rural areas demonstrating higher scores than those from urban areas. Higher number of incidents attended was associated with higher stress levels. Sample size too small to measure statistical significance. | Not discussed/noted or the focus of the paper. | “Need more of a culture to discuss this in the industry as a whole, particularly in the ambulance service. We need to take violence more seriously and not brush over it”.  Participants suggested the following violence prevention strategies: Continued flagging of dangerous addresses, duress alarms, increasing police presence at incidents, enhancing officers’ knowledge in violence minimisation strategies, improved training to deal with aggressive patients (current training is inadequate).  Authors suggest that the needs of patients have been prioritised over the protection of staff. |
| Clompus & Albarran [45]  2016  England | Discussed impact of distressing cases that may lead to compassion fatigue, compartmentalising, or distancing emotionally from the patient.  Organisational stresses and key performance requirements such as quotas, and standards such as response times and expectations as part of the job that must be managed and requires resilience. | Not discussed/noted or the focus of the paper. | Four strategies:   - Organisational strategies such as visits by team leader to see how they are coping - Informal support from peers and use of humour - Detaching and blocking as an individual - External supports came from family and friends or by referring themselves to an outside agency such as their GP |
| Coxon et al. [46]  2016  England | Fatigue, stress, difficulty switching off, high rates of sickness and days absent. Rates twice as high as other healthcare professionals. | Fatigue, poor work-life balance, poor post-shift recovery. | EMDs perceive that paramedics see “us and them” culture. |
| Donnelly & Bennett [20]  2014  USA | Possible PTSD due to exposure to critical incidents. Noted that it can be cumulative or the impact not felt for several months. Hence, the questionnaire asked them to recall as far back as 6 months. Males report more critical incidents and stress than women, but there is no difference in levels of PTSD. | Not discussed/noted or the focus of the paper. | In the qualitative component of the study, participants listed these additional critical incidents to the ones provided in the questionnaire.  Critical incident stress inventory for EMS:   - Encountered a child that had been accidentally killed - Encountered a child that had been murdered - Encountered an elderly person who was severely abused or neglected or in dire need of medical attention because of abuse or neglect - Encountered patient who was severely burnt - Encountered a suicide victim - Encountered a drowning victim - Was assaulted by a patient - Present when a fellow EMT/Paramedic or other first responder was seriously injured; or first responder was killed - Was in a serious accident with an ambulance or other emergency response vehicle |
| Donnelly & Siebert [9]  2009  USA | Can lead to stress-related disorders that have either a mental or physical impact and higher than general public levels of alcohol and drug abuse. Evidence suggests that EMTs’ rate of alcohol and drug abuse accelerates following exposure to critical incidents. Insufficient evidence to comment if subculture has a role in rates and impact.  PTSD has been studied more than other impacts; e.g. depression, other anxiety disorders. Also explored PTSS and suggests a continuum between PTSD and PTSS which acknowledges that symptoms may be the same. | Notes that there is an impact on physical health but does not list any specific outcomes. | Not discussed/noted or the focus of the paper. |
| Dropkin et al. [21]  2015  USA | Not discussed/noted or the focus of the paper. | Major physical issues are musculoskeletal.  Weight of the patient and manual handling.  Blood-borne pathogens.  Needle stick injuries mostly with inexperienced EMTs. | Organisational and psychosocial factors related to incident injuries or illnesses.  Importance of each of the following in relation to developing injuries or illnesses:   - Lack of choice in choosing partner (100%) - Pre-employment screening and agility testing (100%) - Provide referral sources to medical doctors (97%) - New or inexperienced partners (93%) - Provide an effective policy for reporting injuries (89%) - Ambulance design (88%) - Missing scheduled meal and restroom breaks (73%) - No batteries for power stretchers (70%) - Poor organisational social support (69%) - Shift longer than 12 hours (62%) - Maintenance checks on all equipment (48%) - Training, proper resources/assistance in the field (41%) - Second job (35%) - No control over work environment (33%) - Lack of backup support/crew (27%) - Lack of good or updated equipment |
| Flannery [22]  2015  USA | Can lead to PTSD. | Not discussed/noted or the focus of the paper. | Not discussed/noted or the focus of the paper. |
| Forslund et al. [41]  2004  Sweden | Creates stress, anxiety, and uncertainty. | Not discussed/noted or the focus of the paper. | Need for training. |
| Gallagher & McGilloway [50]  2008  Ireland | Critical incidents that are of significance were the death of a baby, suicide, and grotesque mutilation.  Impacts included angry outbursts, sleep problems, recurring dreams and nightmares, an increase in alcohol consumption, feeling alienated from other people, and an inability to relax. Many also described feelings of despondency, intrusive thoughts of the incidents, and flashbacks, while some others said that they had become irrational, and over-protective due to the high levels of stress they encountered in the course of their work.  Impact can be cumulative and/or delayed and lead to long-term sick leave.  EMCs reported lack of control over their work, particularly when had traumatic events and callers got criticised by other first responders (police, fire, and paramedics) and by the public. | Impact of CIS on physical health included weight gain, back problems, and lack of appetite. Many respondents reported it was difficult for them to maintain or improve their general levels of fitness because of shift work and the lack of on-site exercise facilities. Smoking-related illnesses due to an increase in their smoking habits.  Sub-theme related to effect on family relationships: Over half of the interviewees (14/27) mentioned that their personal relationships and home lives had been negatively affected due to work-related stress. Some reported having ‘angry outbursts’ at home after a stressful day, or not being able to talk to their partners about distressing incidents. The long working hours and shift work also reduced the amount of time they spent with their family. | Not discussed/noted or the focus of the paper. |
| Gist & Harris Taylor [23]  2008  USA | Can produce stress and PTSD. | Not discussed/noted or the focus of the paper. | Not discussed/noted or the focus of the paper. |
| Golding et al. [47]  2017  England | Feelings of being out of control.  Physical layout impacts on control.  Lack of control and knowledge of outcomes.  Vicarious trauma and burnout stress.  PTSD results at high levels, and high levels of sick leave.  Strong emotional attachment to their job as a public service. | Not discussed/noted or the focus of the paper. | Need for quality supervision.  Need managers who are closer to them in working relationship.  Conflict between workers not managed by supervisors.  Seek social support from peers, family, and friends.  Used black humour. |
| Halpern et al. [34]  2009  Canada | May lead to PTSD, and in many cases, leads to emotional responses, burnout.  Critical incidents may lead to somatic symptoms (e.g. headaches, gastrointestinal distress), sleep disruption, sadness, avoiding thoughts or situations, intrusive memories, anger at the organisation, irritability, job dissatisfaction, social withdrawal, relationships affected negatively, loss of compassion, and substance abuse. | Mentioned, but not identified. | Need for education, training, supervisor support. |
| Halpern et al. [35]  2009  Canada | Not discussed/noted or the focus of the paper. | Not discussed/noted or the focus of the paper. | Participants highlighted the importance of supervisor support and a timeout period immediately following a critical incident.  Participants provided the following suggestions to help in dealing with critical incidents:  Education for EMTs, supervisors, and families on recognising signs of critical incident stress.  Addressing barriers to support by education about stigma in the workplace and improving supervisors’ capacity to reach out to EMTs.  Improving chronic workplace stressors.  The introduction of morbidity and mortality rounds (non-judgmental format for feedback on difficult cases commonly used in medicine). |
| Hegg-Deloye et al. [36]  2014  Canada | A high proportion of EMS shift workers reported work stress, low job satisfaction, and poor mental health. | Fatigue and poor physical health were reported by a high proportion of EMS shift workers. | Not discussed/noted or the focus of the paper. |
| Hugelius et al. [42]  2014  Sweden | Ambulance personnel face potentially stressful events and regular exposure to traumatic situations that could injure or have an emotional effect on the individual (depression, irritability). Other reactions included reduced compassion or losing their tempers.  These reactions were associated with the lack of crisis support interventions after traumatic events and ambulance staff ‘‘collecting’’ negative emotions and experiences with a limited possibility for mental health recovery. | Not discussed/noted or the focus of the paper. | Not discussed/noted or the focus of the paper. |
| Jonsson & Segesten [43]  2004  Sweden | The structure of a traumatic experience described as a six-fold experience that begins before the traumatic event occurs and ends with handling and recovery.  Pre-trauma experience is characterised by use of an “inner dialogue” for preparing oneself, to prevent stress and increase one’s alertness by visualising what might be faced, even though it may be unknown and therefore impossible to fully prepare for.  Recognising that it could have been their family or themselves as the victims, resulting in feelings of anxiety.  Fear of failing in their responsibilities, they articulate feelings of vulnerability and isolation, and difficulty in distancing themselves from the patient or their family.  Risk of making mistakes and fear of misjudging or failing in their desire to help the patient. Feeling insufficient and worthless even if they have done everything possible to help and support the patient and family.  Be professional and hide their anxiety/emotions and try to convey comfort and calmness. The challenge of containing emotions and not being able to distance themselves (from the event) makes it more difficult to leave it behind.  Before leaving the scene, initial feelings and evaluation of how they are going to cope.  After handover or completion of the scene, they can feel confused, upset, exhausted, sad, and distressed, and that the world is chaotic. Intensity and duration of these feelings varies considerably. The intensity of feelings varied a great deal, from over-powering feelings of chaos to reflection over what had actually happened.  More subtle feelings included being rejected by relatives or fellow workers, feelings of anger, frustration, resentment and bitterness, betrayal and rejection, self-loathing, guilt and humiliation, being out of control, trapped, and feelings of helplessness. Overwhelmed by feelings that they cannot escape from characterised by confusion and chaos, loss of control, and isolation from the world, intrusive memories, and a sense of unreality.  Post-traumatic stress disorder symptoms included: re-experiencing the trauma, numbing of compassion and distortion in social or professional performance, and symptoms of increased arousal. Trying to hide feelings or appearing stoic. For some, feelings and memories were so prevalent that they dominated their normal life. Re-experiencing occurred most commonly in the form of nightmares and flashbacks that continually developed into traumatic memories.  Feelings of guilt, shame, and self-loathing, even if they knew that they did everything possible to help and support the patient.  Most shameful for some participants was not that they failed in caring or giving attention to the patient, but that they were so overwhelmed by all impressions from the scene of the accident. Self-impression that they could work well under pressure and would cope with high levels of stress and that nothing could shake them. Terrified, suffer from sleeping disturbance, nightmares, intrusive memories. Feelings of uselessness, shortcomings, and powerlessness, and how others see them. Strong expressions of isolation and feelings of a lack of understanding from their closest social network. | Participants reported being so overwhelmed by their emotions that they get physical sensations such as feeling sick or fainting during a traumatic situation.  Just being there with the patient in a traumatic situation demands a lot of self-possession and strength that can cause feelings of fatigue. | All the respondents thought that to handle a traumatic experience, it was necessary to have someone (either colleagues or others) to share the worries with and talk to about their feelings. The need for someone to talk to was usually instant and urgent. Some of the participants indicated that it was necessary to hand over all the anxiety to someone who could hold and enclose it. |
| Klimley et al. [24]  2018  USA | Exposure to potentially traumatic calls shown to increase anger outbursts, nightmares, flashbacks, alcohol use. Adams et al. (2015) conducted semi-structured interviews with 16 emergency medical dispatchers to explore their levels of stress and overall well-being. Found that organisational and administrative difficulties exacerbated dispatchers' negative feelings (e.g. powerlessness, failure/lack of control), sleeping difficulties, substance use, emotional numbness, hyperarousal, and isolation). | Organisational and administrative difficulties exacerbated dispatchers’ sleeping difficulties. | Not discussed/noted or the focus of the paper. |
| Larsson et al. [44]  2016  Sweden | It is not only the frequency and intensity of the everyday reactions that matters, but also the personal significance ascribed to what is happening. | Not discussed/noted or the focus of the paper. | Problem-focused coping: differences were observed between different hierarchical levels. At the lower end of the organisations, examples of problem-focused efforts included taking measures to uphold one’s own security and to omit from safety procedures in order not to provoke. Informants higher up in the hierarchy reported strategies like demanding resources from their superiors and developing plans of action.  Emotion-focused coping: favourable thoughts and actions were reported in addition to passive and avoidant strategies. One example involves constructive emotional confrontation, for example seeking information as a sort of feedback on the outcome of a stressful situation. Examples of emotional distancing include to put feelings aside and to not dwell on things, to joke, to change the subject, and to be professional and focus on the task assignment. |
| Lindahl [25]  2004  USA | Years after the critical incident, firefighters/paramedics continue to suffer from PTSD and major depression with suicidal ideation. Symptoms include: terrifying intrusive thoughts, exaggerated startle response, and memories of emergency calls going back throughout career, triggered by such stimuli as the sound of sirens or certain smells; attempts to avoid thoughts by staying at home and in bed, avoiding newspapers, television, and friends; excessive sleep (up to 20 hrs a day); intense and disturbing nightmares; mental confusion and memory loss; halting and stuttering speech; the inability to concentrate or read; and a loss of the experience of pleasure in any activities.  Despite medication, hospitalisation, electro-convulsive therapy and treatment from a psychiatrist/psychologist, treating professionals believe unlikely ever to work again. | Not discussed/noted or the focus of the paper. | Not discussed/noted or the focus of the paper. |
| Mahony [48]  2005  UK | Not discussed/noted or the focus of the paper. | Not discussed/noted or the focus of the paper. | Not discussed/noted or the focus of the paper. |
| Mahony [54]  2001  Australia & UK | Not discussed/noted or the focus of the paper. | Not discussed/noted or the focus of the paper. | Not discussed/noted or the focus of the paper. |
| Paterson et al. [10]  2014  Australia | Not discussed/noted or the focus of the paper. | Not discussed/noted or the focus of the paper. | Not discussed/noted or the focus of the paper. |
| Pow et al. [37]  2017  Canada | Not discussed/noted or the focus of the paper. | Not discussed/noted or the focus of the paper. | Not discussed/noted or the focus of the paper. |
| Pyper & Paterson [29]  2016  Australia | Stressors included treating personally known patients, working with children, critically ill patients, the death of patients, community expectations, ‘office politics’ (including paperwork, colleagues, and management), fatigue, working as a single officer, and longer response times due to travel distances.  Majority of rural and regional ambulance personnel reported normal levels of stress. Working with known individuals in a community may offer some degree of ‘protective’ impact for rural and regional ambulance personnel, at least in terms of stress. | Negative effects of being on-call for healthcare worker’s sleep and clinical performance. | Not discussed/noted or the focus of the paper. |
| Regehr & Millar [38]  2007  Canada | Paramedics in this study perceived the amount of work they were assigned was excessive.  Clear association between control, self-efficacy, and distress.  Paramedics identified the fast pace of work, the excessive amount of work, and the emotional intensity of their work as stressors/work environment as being high in demand, low in control, and low in support. | Interaction between demands of work pace and the psychological demands of dealing with tragic situations, leaving little time for physical rest or psychological processing of tragic encounters. | Not discussed/noted or the focus of the paper. |
| Regehr et al. [39]  2002  Canada | Secondary/vicarious trauma.  Trauma can be cumulative and/or caused by smaller significant events.  Intrusive imagery, generalised fears, sleep disturbances, a changed worldview, and affective arousal – the result of empathic engagement between the worker and the client.  Exposed to at least one of the listed critical incidents during the course of their career, including the death of a colleague, injury on duty, mass casualties, or the death of a child.  Most commonly reported events defined by respondents as traumatic for themselves were suicides and violence against children. In cases of child abuse and neglect, in particular, they were able to recall in specific detail aspects of the victim and environment in which he or she was found.  Dealing with grief/violence directed toward themselves was not described as traumatic.  IES scores reveal that 25.5% of the 86 respondents fell into the severe or high range of post-traumatic symptoms, 14% into the moderate range, while 44.2% into the low range of symptoms.  Depression/alcohol-related problems/arousal symptoms, such as anger and fear; and avoidance symptoms, such as emotional blunting/tearfulness, shortness of breath, flashbacks, and night terrors/exposed worker at times felt disengaged and emotionally distant from family members.  Coping strategies:   - Cognitive focus (conscious process of emotionally distancing themselves and ensuring that they did not become emotionally attached to the victim or the victim’s family) - Thick skin to a lot of the calls, as a protective mechanism - Need to obtain information about the situation after - In order to cope with the lack of control over the job, ensured that other aspects of life were in control and that family was prepared for possible disaster | Not discussed/noted or the focus of the paper. | Reduced capacity to handle stressful events, depression, and substance use. Although many of the responders described the use of alcohol as a short-term coping strategy, there was a recognition that this, at times, became problematic. |
| Rice et al. [30]  2014  Australia | High job demands, intensity in care delivery, job burnout, and loss of staff due to health issues, have resulted in a continuum of health disruption for practitioners.  Prevalence of overweight and obesity reported in healthcare workers; 50% of paramedic’s shift is spent sitting, contributing to obesity.  Stress at the forefront of care delivery, and the effects of stress impacted on participants' physical health. | Increased incidence of musculoskeletal injuries among healthcare workers.  Factors contributing to poor physical health are long hours and shift work, resulting in fatigue and exhaustion, impacting upon clinical decision-making and workplace responsibilities.  Chronic fatigue levels in paramedics are at a peak and were significantly higher than 3 groups of non-­clinical shift workers; opportunities for physical activity are restricted due to roster.  Physical health was perceived as more than the physical capability to perform at work.  Concerned about the quality of their diet because fast-paced work demands compromised their eating habits.  A connection between physical health and job satisfaction to subsequent retention in their profession. Physical health consistently acknowledged as important, albeit expected levels of physical activity were not within recommended guidelines. | Not discussed/noted or the focus of the paper. |
| Roth & Moore [26]  2009  USA | Providers are exposed to high stress situations on a routine basis and often face challenging environmental forces, violent patients, or aggressive bystanders on scene, shifts, overtime. shift rotations, and call volumes.  Negative interactions at work on family functioning; personal coping resources may mediate the impact of stress on the family.  The nature of emergency services work, EMS personnel are particularly vulnerable to the effects of work stress and trauma:   - *Shiftwork, Holidays, and Social Life “Just Not Being Able to Go”* Shift work impacted the individuals and family’s social life at other times of the year and how operating in a “9 to 5 world” could be problematic - *Changes in Marital and Parental Roles: “The Demands of His Job”* Shift work forced changes in family roles; some mentioning adjustments to marital roles and addressing parental roles. Challenges with intimacy - *The Rhythms of Home Life: “There’s No Set Scheduling”* Shift work was disruptive to the schedule and rhythms of home life - “Sometimes I kid with him and I call myself the ‘EMS widow’ because that’s my sacrifice in understanding that he needs sleep” - *Concerns about Physical Safety and Job Risks* | Not discussed/noted or the focus of the paper. | Coping with the Impact of EMS Work on the Family System.  Cope with the impact of EMS work by employing the following - emotional support, positive thinking to manage stressors, negotiating family responsibilities, seeking social support from others, and developing their own interests.  Provision of emotional support to their EMS family member through talking and listening or giving their family member “space” to de-stress as coping strategies.  Cognitive Strategies:  *“Go with the Flow” while “Looking at the Worst Possible Scenario”.*  *Seeking Social Support: “Cultivate Those Friendships”.*  *Negotiating Family Role Responsibilities: “How to Balance Each Other”.*  *Developing Your Own Interests: “I Do Yoga; I’m Also a Writer”.* |
| Skogstad et al. [51]  2013  Norway | PTSD ‘arises as a delayed or protracted response to a stressful event or situation (of either brief or long duration) of an exceptionally threatening or catastrophic nature, which is likely to cause pervasive distress in almost anyone’.  Risk of developing PTSD depends on the nature of the critical incident, the individual’s personality and life history, and events that may occur in the aftermath of the trauma. Social support, mainly emotional support, has been shown to be protective against the development of PTSD. Co-morbidity between PTSD and disorders such as depression, anxiety, and substance abuse.  Ambulance personnel are frequently exposed to critical incidents and generally report more health problems. PTSD prevalence in some studies has been close to 20%. Lack of social support, unacceptable organisational conditions at work, and individual factors have been associated with more PTSD symptoms.  Suffer from persistent stress symptoms as a result of frequent exposure.  Increased risk of being exposed to traumatic events through their daily work. Individual differences in vulnerability and resilience appear to be important factors for the intensity and duration of trauma-related symptoms experienced in the aftermath.  Nature of the trauma is also an important factor; for example, dealing with child victims is known to be particularly distressing. | Not discussed/noted or the focus of the paper. | Not discussed/noted or the focus of the paper. |
| Sofianopoulos et al. [31]  2012  Australia | Continuing growth in the caseload predicted, risk of sleep disturbance, fatigue, normal circadian rhythm and sleep hygiene is continually challenged with increased workloads.  Sleeping problems, headache, and stomach symptoms. Health complaints were associated with the psychological demands of the job.  Scottish ambulance personnel attributed burnout to less job satisfaction, longer time in service, less recovery between incidents, and more frequent exposure to incidents. | Not discussed/noted or the focus of the paper. | Not discussed/noted or the focus of the paper. |
| Sterud et al. [11]  2006  Norway | Ambulance workers have a higher standardised mortality rate, higher level of fatal accidents, higher level of accident injuries, and a higher standardised early retirement on medical grounds than the general working population and workers in other health occupations.  Prevalence of post-traumatic stress symptom >20% in five of seven studies, and similarly high prevalence rates were reported for anxiety and general psychopathology in four of five studies.  Prevalence of PTSD symptoms is consistently high. | Job conditions and psychological demands were significantly associated with more sleeping problems, headache and stomach symptoms, and problems with the neck, shoulders, and knees. In a study from the UK, ambulance workers (n = 52) reported more physical health problems on average (15.1 v. 13.8) than the general working population. | Not discussed/noted or the focus of the paper. |
| Varker et al. [32]  2018  Australia | Organisational factors are the most commonly researched as they relate to mental health problems and/or well-being. | Not discussed/noted or the focus of the paper. | Not discussed/noted or the focus of the paper. |
| Wiitavaara et al. [40]  2007  Sweden | Vulnerability, and a feeling of helplessness, when witnessing the horrible scenes.  Situations involving children were always perceived as hardest to handle.  A balance between becoming too vulnerable and becoming hardened. | The work of ambulance personnel involves a heavy physical workload and strain of irregular hours. Situations that demand instant alertness, working under time pressure, handling severe situations involving human suffering and death.  Described bodily illness mainly from the lower and upper back and from the neck/shoulder region, but also from the knees and wrists. | Striking a balance.  The experience of illness and wellness were considered part of the work. Accepting and handling the present illness was important to their framing of what wellness meant.  Sub-themes were: Attaining and maintaining wellness through nurturing; Encountering illness as an experience and a threat; and Accepting and handling illness.  Wellness was nurtured by the experiences of *Getting excitement and being challenged; Having freedom and flexibility; Being ‘‘someone’’ and making a difference; and Being one of the gang*. Self-determination to make one’s own decisions, build rapport with the individual patient and provide individually-tailored care were crucial to wellness. Flexibility in work environment and tasks.  Being ‘‘someone’’ and making a difference was a third aspect of nurturing and being nurtured. Being ‘‘someone’’ meant being visible, centre stage, as it were; being in the limelight. It also meant being the rescuer, the hero, in a crisis situation. Uniform as a part of identity and making a difference to others.  Illness can be encountered as an experience and a threat. The illness experience had a physical, psychological, and social dimension encapsulated by:   - The body makes itself heard - One can get worn out - One can become too vulnerable or hardened |
| Wolkow et al. [33]  2015  Australia | Paramedic personnel in Australia were found to have a higher prevalence of sleep-related mental health outcomes (i.e., depression and anxiety). Evidence suggests that an increase in stress exposure simultaneously induces both physiological (i.e., higher & flatter diurnal cortisol levels and/or abnormally high or low cytokine levels) and psychological changes (i.e., mood & behavioural disturbances), and that these responses can be positively or negatively correlated with one another.  Depressive symptoms increased as subjective sleep quality deteriorated. | Periods of partial and total sleep deprivation/restriction can impair immune function (e.g. above and below normal pro- and anti-inflammatory cytokine levels), hormone secretion (e.g. higher & flatter diurnal cortisol levels) and instigate adverse psychological changes (e.g. symptoms of anxiety & depression).  Inadequate or disrupted sleep associated with cardiovascular, metabolic diseases and depression.  Elevated levels of sleep regulating cytokines interleukin (IL)-6, IL-1β and TNF-α have been positively associated with CVD, metabolic syndrome, and depression. Higher, flatter diurnal cortisol patterns have been related to depression.  Elevated morning cortisol levels measured in plasma have also been positively associated with CVD and metabolic syndrome-related features (e.g., glucose intolerance, insulin sensitivity, hypertension, atherosclerosis).  Both increases and decreases in cortisol level have been demonstrated following stress exposure and could indicate allostatic load (i.e., wear and tear) on the endocrine system expressed as either an intensified or suppressed cortisol production.  Sleep restriction studies have demonstrated an increase in daily cytokine levels. Pro-inflammatory cytokines IL-6, IL-1β, IL-1ra and TNF-α significantly increase or decrease from baseline following single as well as multiple nights of complete and partial sleep restriction. | Not discussed/noted or the focus of the paper. |
