# Supplementary material for: The effects of emergency medical service work on the psychological, physical, and social well-being of ambulance personnel: a systematic review of qualitative research
Source: BMC Psychiatry. 2020 Jul 3;20:348. doi: 10.1186/s12888-020-02752-4 (PMC7332532; doi:10.1186/s12888-020-02752-4)
Supplement: Supplementary file 4 — Additional file 4: Appendix 4. Impacts of organisational and systems parameters [file 12888_2020_2752_MOESM4_ESM.docx]

**Appendix 4: Impacts of organisational and systems parameters**

| **Author, year, country** | **Question 4: Effects of workflow & nature of work on mental health and well-being** | **Question 5: Effects of organisational structures on psychological and physical well-being** |
| --- | --- | --- |
| Adams et al. [27]  2015  Australia | When Emergency Medical Dispatchers (EMDs) can follow-up with paramedics to get closure/find out what the outcome was, both were better at coping with events. | Including EMDs alongside paramedics in debriefs helped them feel valued, have psychological closure, and feel heard and understood by each other.  EMDs rarely got positive feedback from managers and paramedics or the service; only feedback when something goes wrong.  The dispatcher role is often to enforce unpopular management rules and regulations, which creates division among the service types. |
| Alzahrani et al. [52]  2017  Saudi Arabia | Not discussed/noted or the focus of the paper. | This paper noted that the third intervention when the referral process is directly between the employee and the counselling service within 6 weeks numbers increased. |
| Avraham et al. [53]  2014 | Not discussed/noted or the focus of the paper. | Not discussed/noted or the focus of the paper. |
| Bledsoe & Barnes [19]  2003  USA | Not identified, although suggests most EMS stress is about the organisation, not trauma incidents. | Not identified. Suggests that critical incident stress debriefing is harmful and should not be part of an emergency organisation’s strategies for dealing with minor or major trauma. Suggest using psychological first aid as a strategy.  Suggest psychological first aid*: listening, conveying compassion, assessing needs, ensuring basic physical needs are provided, not forcing talk, providing or mobilising family or significant others, protecting from additional harm* (p 66). |
| Bracken-Scally et al. [49]  2015  Ireland | One of the most distinctive characteristics of emergency service work is the reality of regular exposure to trauma and a range of other work-related stressors. | Employers have a duty of care to protect their staff against the effects of stress and trauma. Arguably, this duty of care also applies to employees who are about to retire, or who have just retired, from high-risk occupations. |
| Chappell & Mayhew [28]  2009  Australia | Not discussed/noted or the focus of the paper. | Not discussed/noted or the focus of the paper. |
| Clompus & Albarran [45]  2016  England | The change in the healthcare system culture was explored several times in this paper and described as the increased pressure to achieve targets (time to job, time at scene, time dropping patient off at hospital), changes in the skill mix and roles (introduction of emergency care assistants, and single manned rapid response vehicles and the use of standby points) seen to have eroded team support and opportunity for peer feedback. | Paramedics said they rarely get positive feedback from managers.  No comment was made on the effect of informal structures that addressed respite. |
| Coxon et al. [46]  2016  England | Stress caused by too few call-takers and dispatchers, and at the other end, not enough ambulances, which left the dispatcher waiting with a very sick patient.  Training was seen as inadequate - done in multi-disciplinary team with paramedics did not seem relevant. | EMDs said they rarely get positive feedback from managers and paramedics or the service; only feedback when something goes wrong.  Significant time commenting on poor training, too little training and education.  Poor interpersonal relationships with paramedics on the road and with other staff.  Breaks during the day were often not taken as, when this was done, it was difficult to get a sense of subsequent jobs.  Strategies: training, going out on-road, separating out work from home, and talking to partner.  Strategies for mentally winding down between work and home – post-work strategy for letting go. |
| Donnelly & Bennett [20]  2014  USA | Not discussed. | Not discussed. |
| Donnelly & Siebert [9]  2009  USA | The literature review identifies 2 sources of stress: workplace stressors and critical incident stressors. Workplace stressors are: 1) insufficient salary; 2) an alienated and unsupportive administration; 3) lack of support from, or conflict with, colleagues; and 4) interference with non-work-related activities.  Added to these are risk of exposure to pathogens, threats of both verbal and physical violence, injury, and death due to vehicle crashes, and in the USA working 24 hours shifts, along with management lack of support, conflict with peers, etc.  Second source is critical incidents - issues to do with patient care.  Mitchell's initial conceptualisation of critical incidents included:   - The serious injury or death of an emergency team member in the line of duty - The serious injury or death of a civilian resulting from emergency service operations - Cases charged with profound emotion, such as the death of an infant - Cases that attract unusual attention from the news media - A loss of life after a prolonged rescue effort - Serious physical or psychological threat to the rescuers - Incidents that surpass the normal coping mechanisms of personnel   The initial conceptualisation of critical incident stress is formulated in a broad enough way to encapsulate most critical incidents. However, it would be difficult to quantify or measure "incidents that surpass the normal coping mechanisms of personnel”.  Added stress in dealing with acutely ill or seriously injured people, dealing with psychiatric patients, dealing with family and friends, dealing with dead bodies.  Critical incidents have a cumulative effect.  Uses the term binary - workplace stressors can be intermittent, but also cumulative. | Does not deal with this directly, except to identify the workplace as a stressor.  Protective factors are categorised under social environment and personal resources:  *Social environment*  Social environment stressors include an alienated and unsupportive administration, a lack of support from or conflict with colleagues, and occupational interference with non-work-related activities.  The paramilitary and cohesive environment of the service is seen as supportive – positive social relationships within the services, bonding with peers, and bonding vertically with managers.  *Personal resources*  Personal resources are divided into two sections: 1) Demographic characteristics (e.g., age, gender, race, level of training, years of professional experience); and 2) personal psychological factors (e.g., cognitive structure and coping mechanisms - having internal locus of control).  Training programs that develop internal locus of control seen as helpful in developing stress coping skills. |
| Dropkin et al. [21]  2015  USA | Shorter shifts associated with reduction in fatigue and injuries; however, staff prefer longer shifts so they can work a second job due to low pay.  Staff wanted third partner to assist with load.  Equipment and ambulance contributed to physical injury.  Lack of consultation contributed to injuries. | Discussion with organisation on needs would assist with better equipment and working conditions.  Suggestions for improvement include:   - Better equipment - More resources - Discussions with EMT on-road - Third person on vehicle - Opportunity to exercise/walk between jobs - Opportunity for proper meal breaks to eat healthy food |
| Flannery [22]  2015  USA | Not discussed/noted or the focus of the paper. | The suggestion is for a multi-modal response by therapists to paramedic stress.  Author demonstrates that single therapy interventions such as CBT have been shown to be ineffective for first responders.  Clinicians treating rape and disaster victims have created integrated treatments, but these have not been trialled with first responders. This may be due to a lack of media focus on first responders.  First responders go from one critical incident to another, so limited time between them.  Little time for counselling.  First responders may self-medicate to deal with issues.  First responders do not have links to services.  Trauma disruption to three domains: mastery over environment, caring attachment to others, and meaning and purpose.  Psychological symptoms include memories of the event, avoidant symptoms, and reduced interest in life activities.  Approaches to mastery: Limit exposure time onsite, update local responders on their families, field workers from outside the area. These strategies are made difficult by the organisational culture.  Caring attachment: social support sometimes helpful.  Meaning: CBT has been found to be effective in restoring meaningful purpose.  Symptoms: CBT does not always resolve symptoms. Medication and mindfulness may work.  No studies have examined in vivo de-sensitisation procedures.  Need studies to research multi-modal approaches that are rigorous in design. |
| Forslund et al. [40]  2004  Sweden | Work is uncertain: Information is diffuse and limited and they often are not aware of context.  There can be communication difficulties when dealing with a child or hearing-impaired person, when medical language is used or speaker is a second language speaker. | Not discussed/noted or the focus of the paper. |
| Gallagher & McGilloway [50]  2008  Ireland | Lack of time for recovery given workloads.  Lack of concern from management.  Poor rosters.  Failure of management to recognise impact of critical incidents.  Need more training and education in stress management, especially for new recruits.  Need recovery time - not provided.  Supervisors to recognise signs of stress.  Call-takers work alone at night and on weekends when it can be busy. | Lack of time for recovery, given workloads.  Lack of concern from management.  Poor rosters.  Failure of management to recognise impact of critical incidents.  Need more training and education in stress management, especially for new recruits.  Need recovery time - not provided.  Supervisors to recognise signs of stress.  Call-takers work alone at night and on weekends when it can be busy. |
| Gist & Harris Taylor [23]  2008  USA | General workplace conditions impact on stress levels.  Consideration needs to be given to HR components, e.g., worker’s compensation, employee assistance, health benefit plans, as well as operational practices and protocols. Need a process within the organisation that links essential HR operations to management practices, and provides structure that allows them to be applied when necessary. | Essential for both formal and informal approaches to be offered.  Suggests that, for the most part, informal strategies are effective.  Critical incident stress debriefing is not necessarily the best approach, and in the US context, it is sometimes done by untrained or poorly trained practitioners. |
| Golding et al. [47]  2017  England | Training inadequate resulting in increased stress and a negative perception of the organisation, and lower personal responsibility for performance.  Organisation of work impacts on stress; e.g., supervisor’s lack of skill.  High workload and lack of control over their work. | Not directly noted.  Strategies to cope with negative effects of workplace stressors included use of emotional regulation and engaging in peer support. Some evidence from this review suggests the work environment does not always provide opportunities for sufficient peer support.  Existing research from other emergency medical settings suggests that the peer support element of working in a team can be beneficial for managing stress and preventing burnout.  Interventions in the form of cognitive behaviour therapy or mindfulness. |
| Halpern et al. [34]  2009  Canada | Definition of critical incidents.  Ambulance workers appeared to use a working definition of critical incidents as a workplace phenomenon which they differentiate from chronic workplace stressors.  Critical incidents are discrete incidents in the field which are attended by the ambulance worker. They involve strong emotions which last for long enough to be uncomfortable in their own right or to produce uncomfortable sequelae. The emotions were often sadness or anger.  Chronic workplace stressors include high volume of workload, ongoing difficulties with management, and shift work. These may impact on the experience of critical incidents, but they are also distinguished from it.  The management of the incident by the organisation before, during, and after it has transpired are considered an important aspect of the incident. They regard critical incidents as an often distressing component of their occupation which should be managed primarily within the organisational context. | Not identified directly, although strategies for managing it included.  Peer and supervisor support, as well as a brief period post-incident in which to access them.  A significant barrier to accessing support in the workplace was their difficulty in acknowledging distress.  The culture of the organisation stigmatises vulnerable feelings. However, more recent recruits tended to feel quite comfortable with their own feelings of distress. |
| Halpern et al. [35]  (2009)  Canada | Supportive qualities demonstrated by supervisors included:   - Acknowledging the incident as critical (implicit or explicit) - Expressing concern about the well-being of the EMT - Willingness to listen - Valuing the EMT’s work - Offering material help   Barriers for EMTs requesting support from supervisors included:   - Fear of stigma and appearing weak - Not recognising the call as a critical incident - Avoiding thinking or speaking about the call, or just saying “I’m fine” - Expecting an unsupportive response   Barriers for supervisors in providing support to EMTs included:   - Supervisor’s own emotional discomfort - Supervisor’s difficulty identifying idiosyncrasies that make a call critical - Supervisor’s difficulty in recognising an EMT is emotionally affected by a call - Inadequate skills and possibly poor training - Restrictive role definition (e.g. EMTs assuming they‘ve done something wrong if a supervisor approaches them)   Supervisors who were seen as unsupportive were felt to be critical, and were often described in angry, resentful, and disappointed tones. | *Timeout*  Optimally 1/2–1 hour, in which the EMT is taken out of service to spend with peers, and less often alone, e.g., getting a coffee with your EMT partner before starting paperwork, a supervisor took EMTs out of service to have lunch and talk amongst themselves (not necessarily about the call) to relax before going back on shift.  Barriers to timeout:   - Fear of stigma and appearing weak - Time pressures - Concerns about confidentiality - Talking about incidents the EMT has been trying to suppress - Not recognising the call as a critical incident - Avoiding thinking or speaking about the call, or just saying “I’m fine” - Expecting an unsupportive response   *Debriefing*  Perceptions ranged from helpful to harmful. Emergency physicians were sometimes consulted during this early period for reassurance about management of calls involving patients who succumbed to their illness/injury.  New recruits may be hesitant to participate in debriefing if they have not yet developed a strong connection with peers, or who are fearful of appearing weak or incompetent. Often family and friends are the preferred companions for these employees; however, this often has to be postponed until the end of shift. However, more experienced EMTs often protected family and friends from the ‘burden’ of hearing about critical incidents.  Despite the majority of EMTs preferring that interventions be optional, some suggested the most common types of critical incidents (e.g., those involving children) be routinely followed up by supervisors or mental health staff, and even recommend that debriefing be mandatory.  Types of interventions valued by EMTs differ appreciably from the format of Critical Incident Stress Debriefing. Although CISD focuses on the role of the workplace, it is a formal process that is often designated to mental health professionals rather than to the members of the EMS organisation. |
| Hegg-Deloye et al. [36]  2014  Canada | Not noted. | Not noted. |
| Hugelius et al. [42]  2014  Sweden | Managers stressed the importance of an individual perspective and of an awareness that a specific situation might be experienced as a traumatic event for one individual but not for others.  *“It doesn’t have to be traffic accidents or sudden infant deaths”.* | The lack of crisis support interventions after traumatic events were associated with staff “collecting” negative emotions and experiences with a limited possibility for mental health recovery.  The interest in crisis support interventions at a higher strategic level in the ambulance services organisations was fragmented; some managers thought that the issue was a high priority, while others found it difficult to find time and money to perform crisis support interventions.  Ensuring appropriate crisis support interventions after a PTE was seen as an important part of the ambulance manager's role. The challenges described were to identify when, and for whom, crisis support interventions were needed.  Participants expressed doubts about whether mandatory crisis support meetings were the best way of supporting their staff, suggesting that an informal “ordinary cup of coffee and a chat” can be just as effective. The ambulance managers expressed a fear of ‘‘overdoing’’ the supportive approach and of overdramatising the reactions among the ambulance personnel, which could lead to non-supportive interventions.  Additional crisis support intervention info:  Situations in which crisis support interventions had been implemented in recent years included:   - Suicide by a young person - Sudden death of an infant - Aircraft accident - A person who was hit by a train - Situations involving violence or threats against the ambulance personnel   Crisis support interventions were described as single-session group meetings with all involved staff, where they could express how they acted at the scene, as well as their feelings and emotions about the situation. The sessions were led by a designated leader (often the ambulance manager or a senior colleague) and followed a predefined structure. The ideal time to conduct the sessions differed, with responses including: as soon as possible, later on the same day, and the morning after.  All ambulance managers stated that the best option for the staff involved was to stay at the workplace after participation in the crisis support intervention. A few ambulance managers also mentioned practical support as a kind of crisis support intervention, such as the provision of new clothes, or getting in contact with family or friends. |
| Jonsson & Segesten [43]  2004  Sweden | Not noted. | Not noted. |
| Klimley et al. [24]  2018  USA | Not discussed/noted or the focus of the paper. | Not discussed/noted or the focus of the paper. |
| Larsson et al. [44]  2016  Sweden | Not discussed/noted or the focus of the paper. | Favourable informal supports include a supportive climate promoting discussions with colleagues about emotions, and to sometimes socialise outside of work. |
| Lindahl [25]  2004  USA | Not discussed/noted or the focus of the paper. | A firefighter/paramedic was denied Workers’ Compensation following a critical incident as he had previously reported some PTSD symptoms prior to the incident, and PTSD was compensable only as an injury by accident arising from a single critical incident. He then filed an application for a hearing before the Workers’ Compensation Commission who upheld the previous decision.  In a second claim, he alleged that his PTSD was an occupational disease arising from his employment; however, this was denied on the basis that the PTSD resulted from cumulative or repetitive trauma. The firefighter/paramedic then appealed to the Virginia Court of Appeals which reversed the denial of benefits, ruling that the PTSD was an occupational disease.  The employer unsuccessfully appealed the decision to the Virginia Supreme Court who remanded the case to the Court of Appeals, directing it to remand the case to the Workers’ Compensation Commission to calculate the amount of benefits to be paid. |
| Mahony [48]  2005  UK | A reduction in the number of crews, meaning crews from any station can now be deployed anywhere in the large region covered by the service.  The CEO has a long-term plan of seeing each crew and their vehicle as a mobile office/station, forever ready on the highway to respond to situations quickly.  Work has intensified due to the increase in the number of ‘calls from the general public’, meaning that they are sometimes getting called to the next job before they have finished the current one.  Gaps in the roster caused by sickness, pregnancy, recreation leave, and other absences have to be covered by other crew members on a time-in-lieu basis as paid overtime and penalty rates have been traded-off for marginally better rates of pay. However, officers were unable to take time-in-lieu owed to them due to high absenteeism. They did not want to ‘let the team down’ and are expected to show loyalty by giving their time to the service.  Ambulance officers reported heavy workloads and the lack of opportunities to take the agreed-upon two half-hour breaks back at the station in every 12 hour shift, because they were constantly allocated assignments by staff in the Control Room. Relief crews are not assigned or are unavailable because of high absenteeism.  2 senior managers pre-empted complaints about missed meal breaks:   - 1 was empathetic and acknowledged the increased workload on his staff. He related how Control Room managers did not have a crew available to relieve a crew for their meal break back at the station - 1 considered the complaints to be unfounded, commenting that, ‘when I was on the road, there must have been something wrong with you if you couldn’t find time to eat’   Officers worried about low blood sugar causing poor concentration and deterioration in their tolerance levels. | *Less worker autonomy and more management control as stressors*  Personnel exercise little autonomy in regards to administrative or clinical decisions, degrading the skills of experienced ambulance officers.  A satellite tracking system and a computerised data entry system have been implemented. Managers in the control room, rather than the officers, now decide how much time each case should take and when a crew is ready for another assignment. Officers have lost what little control they had over pacing their work and deciding on their recuperation periods to informally debrief with colleagues following a job.  Increased staff surveillance effectively does away with ‘foreign orders’ (crews taking time out to attend to personal activities). Managers would sometimes turn a blind eye’ to ‘foreign orders’ to give officers some sense of control over the pace of their work. Increased staff surveillance will prevent this.  The new technologies are not acknowledged as new means of staff control. They are instead rationalised as being in the interests of accountability and professionalism. If officers resist, they risk being accused of not putting their patient first, of not caring about their patient.  The annual staff turnover rate is only two per cent. The occupational stressors that ambulance officers experience, it seems, are preferable to unemployment stressors.  Decision-making remains centralised in the hands of a few senior executives and not devolved to the ‘road face’ as originally intended.  Each Station Officer has a series of administrative tasks to complete each day, often in their own time as they are on the road all day.  The combination of difficulty sleeping (due to needing time to wind down after a shift) and hunger was said to impact on personal relationships. Additionally, missed meal breaks/irregular meals were associated with gastrointestinal problems and low blood sugar causing poor concentration and deterioration in their tolerance levels.  *The infinite expansion of shift times*  Less staff for each post and longer shifts per member now mean that the service does not need to employ as many people, nor do they need to allocate as many relief positions.  Now that a crew can be sent anywhere in the area covered by the service, it takes longer for officers to get back to their station at the end of shift, causing them to work well over the end of shift.  The indeterminate nature of finishing times makes it difficult for officers to participate in social activities and family life.  Officers were commonly allocated to a job near the end of their shift, meaning that they do not finish until many hours after they were scheduled to.  Staff in the Control Room must send the nearest available ambulance to minimise response times (quality control criteria), not only to improve patient outcomes, but to give the organisation a competitive edge when tendering for contracts.  Rural officers had the added stressor of being ‘on call’ on their days off. They cannot relax with an alcoholic drink nor venture far from home.  Although the ambulance officers of Service UK are supposedly being paid at a rate that is marginally better than the base rate of most other services, officers with small children and a spouse who is not in paid employment have the added stressors of not having the requisite finances for a normative standard of living. |
| Mahony [54]  2001  Australia & UK | *Problems with management*  Problems with management related to control; staff believing that all management decisions, directives, and moves were out of their sphere of influence; powerless to change things.  Described Australian managers as indecisive, failing to communicate plans and decisions with staff. Staff often subject to arbitrary power exercised by managers who created new rules to suit themselves.  UK managers described as organised and efficient; however, this came at the expense of problem-solving and participatory decision-making.  Management’s objectives on efficiency and response times in conflict with the paramedics’ objective of providing quality patient care. Paramedics reported being unable to do this if they are fatigued, rushed, and forced to cut corners trying to meet management’s objectives.  Cumulative impact of stressors: being called to another job before finishing the current one, not having had a break or eating, physical impact (back problems), and colleagues on sick leave.  *Clinical frustration*  Frustration at having to transport clients who were clearly not in need of hospital care, and not allowed to decide which patients are transported to an emergency department; resulting in many unnecessary and inappropriate transfers.  Officers reported being “stuck” at their current level; applications for clinical advancement repeatedly declined; officers described their position as a “dead end job”.  Intense competition to gain entry into the elite paramedic course in the Australian service, creating rivalry where there was once camaraderie. Selection was unfair and marked by favouritism and cronyism. Applying for clinical skills advancement was stressful and demoralising.  Sickness, pregnancy, or recreation leave had to be covered by other ambulance personnel on a time-in-lieu basis, as paid overtime and penalty rates had been traded off for marginally better rates of pay in individually bargained enterprise agreements. All participants had accumulated many hours’ time-in-lieu, which they were unable to take because of the high absenteeism at the time.  For UK participants, it was common to be allocated a patient transfer half an hour before the end of a twelve-hour shift and not to complete an assignment and return to the station until sometimes 7 hours after their shift was meant to finish.  In both services, officers complained that management did not care about their staff; senior managers were considered too distant to know what an officer’s work entailed; road staff felt that they were constantly pushed to achieve more with less resources.  When officers complained to management about working long past end of shift, they were told to *“get on with it, you’ve got a three-day break”.*  Having a founded or unfounded complaint made against them by a member of the public was a stressor, often due to the way senior managers dealt with the situation, causing officers to take sick leave, stress leave, consult mental health practitioners, be on medication and contemplate resigning from the service. Both UK and Australian respondents reported receiving understanding and support from their immediate colleagues and/or Station Officer; whereas, senior managers were considered to be aloof and insensitive.  Australian participants felt that they were not allowed sufficient time between a traumatic assignment and the next case to take part in a debriefing and to recover, and not allowed time to participate in debriefing being held with other emergency services and health professionals involved in the case.  UK services have a long-term plan of seeing each crew and their vehicle as a mobile office/station, continually on the road in order for crews to be located and deployed more quickly.  Australian officers reported other emergency services were better organised to deal with critical incident stress. | Not discussed/noted or the focus of the paper. |
| Paterson et al. [10]  2014  Australia | Paramedics identified the following organisational reasons for fatigue:   - Working time (long night shifts and an inability to rest during night shifts, inconsistent/late/no breaks, working beyond their specified shift finish time) - Sleep (lack of sleep or difficulty sleeping adequately before shifts, and not enough rest periods between shifts) - Workload (high/excessive workload, understaffing) |  |
| Pow et al. [37]  2017  Canada | In addition to the options that participants could choose from on a checklist, occupational stressors reported via open-ended responses included patient care (e.g., pulseless patient with return of pulse, road rage incident, cardiac arrest), issues with coworkers (e.g., partner’s driving ability, incompetence of partner) and working conditions (e.g., lack of resources, missed lunch break, scheduling issues). |  |
| Pyper & Paterson [29]  2016  Australia | Short recovery time between emergencies and frequent exposure to emotionally traumatic events has been shown to lead to burnout as well as a decline in job satisfaction.  Ninety-three percent of respondents reported sleeping 5 h or less while on call, and a further 43% reported sleeping less than 8 h while not on call.  Over half of the participants (55.9%) reported experiencing fatigue at work (35% severe; 20.9% mild). Diverting ambulances to high-volume centres in Australian rural and regional areas has resulted in longer transport times and decreased ambulance availability. | Suggestion to manage sleep and fatigue - changes to the scheduling of on-call periods, ‘protected’ sleep time during on-call periods, or education about fatigue management strategies for on-call periods.  Strategies to manage extended commutes and increased workload as a result of decreased ambulance availability are critical in order to mitigate stress, and contribute to necessary improvements in recruitment and retention in rural and regional areas. |
| Regehr & Millar [38]  2007  Canada | Poor relationships with management, not being valued for their skills, and shift work were the major stressors encountered.  Paramedics reported situations where their skills and knowledge were either not recognised or not utilised.  Paramedics were not given the opportunity to use specialised skills that they had invested time and money into learning.  Lack of resources and high workload/equipment.  Lack of support if legal or clinical audit occurred. | Traumatic exposure is a clear stressor in the work of paramedics; it is the emergency service organisation itself that causes the greatest degree of distress in personnel.  Primary mediating factor of organisational stress is social support within the organisation, particularly from superiors - work group support and more positive supervisor behaviour resulted in lower work-related stress scores, and subsequently, lower rates of psychological distress.  Paramedics have little influence over what should be done at work and how it should be done.  The hierarchical structure of the organisation prevented the ability to influence decisions/authoritarian.  Lack of decision-making authority when trying to admit their patients to hospitals.  Little control over their continued employment, resulting in a fear of losing their jobs.  Feeling unsupported when their supervisors questioned why they were reacting to an event that they did not perceive as traumatic. |
| Regehr et al. [39]  2002)  Canada | Not discussed/noted or the focus of the paper. | The majority of paramedics indicating that they received little or no support from their employers and unions.  Peer support valued/helpful/benefits of “sharing tales” / telling jokes.  The “macho atmosphere” dissuaded workers from discussing concerns and fears.  Events that are flagged by members of the organisation as the ones where assistance is automatically offered are not necessarily the events having the largest impact on workers. |
| Rice et al. [30]  2014  Australia | While participants regarded the importance of physical health as their own responsibility, they believed support in the workplace was also needed. | Key areas that affect retention are job satisfaction and the workplace environment. Professional invalidation, low wages, and limited career progression have also intensified problems for paramedics.  Physical health and the stress of long working hours were intertwined for paramedics. |
| Roth & Moore [26]  2009  USA | Not discussed/noted or the focus of the paper. | Not discussed/noted or the focus of the paper. |
| Skogstad et al. [51]  2013  Norway | Not discussed/noted or the focus of the paper. | Three main strategies to prevent psychological distress following a traumatic event developing into mental illness: pre-employment selection, training in stress management, and early intervention.  Limited research into the effect of pre-employment selection on levels of injury or disease.  Training in stress management is important for personnel who have a high risk of encountering traumatic stress.  Intervening early with psychiatric treatment, mobilisation of the internal resources of the company, is more effective in preventing psychiatric work disability than utilisation of the ordinary healthcare system.  In the acute phase following the traumatic event, it is important that the individual regains emotional control, restores interpersonal communication and group identity, regains a sense of empowerment through participation in work and strengthens hope, and the expectation of a recovery workplace crisis management plan with a trained team will ensure that employees receive information and support which can aid their recovery and help rapid return to work. |
| Sofianopoulos et al. [31]  2012  Australia | Burnout was attributed to reduced job satisfaction, longer time in service, less recovery between incidents and more frequent exposure to incidents.  A relationship between work and mental health is evident, with work impacting the emotional well-being of ambulance personnel.  Occupational stress was a common theme, where paramedics felt unhappy, stressed, and experienced low job satisfaction and poor mental and physical health.  Some research has found no adverse effects of shift work on performance. One paper examining air medical personnel working 24 h shifts revealed that crew members completed the average duty cycle with little sleep debt.  Fatigue resulted in the loss of life and posed a threat to paramedic and public safety (sleeping at the wheel, drug administration). | Scheduling and sleep are important factors. It has been found that shifts following a clockwise rotation are less disruptive to the circadian rhythm (i.e., morning, afternoon, and then night).  Napping before, during, and after a night shift lowered levels of fatigue, increased performance, and reduced diminished performance. |
| Sterud et al. [11]  2006  Norway | Degree of exposure, peri-traumatic dissociation, fewer years of experience, external locus of control, and poor social support predicted higher levels of symptoms.  Significant correlations between frequency of incident stressors, degree of organisational stress, degree of operational hassles, degree of emotional demands and poor communication, length of review following a critical event resulting in loss of life, and PTSD symptoms.  Causes of (early) retirement were musculoskeletal, circulatory and mental disorders. | Not discussed/noted or the focus of the paper. |
| Varker et al. [32]  2018  Australia | Factors relating to workplace mental health/well-being include operational aspects (e.g., shift work, potential occupational risks, job demands); aspects of the individual’s career (e.g., student compared to later career workers) and aspects related to emotional resources, or lack thereof, within the workplace (feelings of workplace belongingness; perceived workplace support and sense of camaraderie; and bullying). | Least researched areas were psychological factors relating to work injury, and intervention studies targeting mental health or well-being.  Few studies in the following areas suicide, personality, stigma, pre-employment factors that may contribute to mental health outcomes, and the use of e-health. No studies were detected which examined the prevalence of self-harm and/or harm to others, bullying, substance use, and barriers to care. |
| Wiitavaara et al. [40]  2007  Sweden | Unwanted organisational re-structuring and the following turbulence and conflicts with increasing demand and educational requirements were described as irritation, frustration, anger, nagging worry, insecurity, lowered self-esteem, lowered status and offended pride, and few possibilities to influence this situation. | Getting relief by sharing experiences with colleagues as part of their daily work routine and by formal debriefing. |
| Wolkow et al. [33]  2015  Australia | Early start times and shift work can cause misalignment to the circadian rhythm of physiological functions. Extended work hours, long commutes, overtime, being on-call can also disrupt sleep. Exposure to environmental (e.g. light and noise), physical (e.g. intense physical work), and/or psychological (e.g. critical decisions, life-threatening situations) work-related stressors can disrupt circadian rhythm.  Extreme sleep restriction over multiple days of emergency work (e.g. 1–7 h of sleep over 2–7 days) can:   - Disrupt the circadian cortisol rhythm - Disrupt (i.e. above & below baseline or control levels) pro- (i.e. IL-6, TNF-α & IL-1β) and anti-inflammatory cytokine levels (i.e. IL-1ra) - Elicit adverse psychological responses (i.e. deterioration in mood) - Cause a simultaneous increase in both cortisol and cytokine levels (i.e., IL-6) | Further investigation is needed to determine, more specifically, the amount and/or number of recovery sleep(s) required for hormonal and inflammatory markers to recover following various types of emergency work. |
