# Supplementary material for: The effects of emergency medical service work on the psychological, physical, and social well-being of ambulance personnel: a systematic review of qualitative research
Source: BMC Psychiatry. 2020 Jul 3;20:348. doi: 10.1186/s12888-020-02752-4 (PMC7332532; doi:10.1186/s12888-020-02752-4)
Supplement: Supplementary file 5 — Additional file 5: Appendix 5. CASP quality ratings of primary research and non-systematic literature reviews [file 12888_2020_2752_MOESM5_ESM.docx]

**Appendix 5: CASP quality ratings of primary research and non-systematic literature reviews**

| **Author, year** | **Aims clearly stated** | **Qualitative methodology appropriate** | **Research design appropriate** | **Recruitment strategy appropriate** | **Data collected in a way that addressed research issue** | **Relationship between researcher & participants adequately considered** | **Ethical issues considered** | **Data analysis sufficiently rigorous** | **Clear statement of findings** | **How valuable is the research** |
| --- | --- | --- | --- | --- | --- | --- | --- | --- | --- | --- |
| Adams et al. [27]  2015 | ✓ | ✓ | ✓ | ✓ | ✓ | ✕ | ✕ | ✓ | ✓ | Excellent interesting paper. Themes focused on operational stress and vicarious trauma arising from coping strategies, organisational stress, and then post-traumatic growth. |
| Alzahrani et al. [52]  2017 | ✓ | ✓ | ✓ | ✓ | ✓ | ✕ | ✕ | ? | ✓ | Perhaps useful for pointing out that privacy and confidentiality are the key. |
| Avraham et al. [53]  2014 | ✓ | ✓ | ✓ | ✓ | ✓ | ? | ✓ | ✓ | ✓ | Useful themes that looked across the ‘on the way’, during, and after the event. Important and detailed analysis and description of processes of control and detachment as coping strategies. |
| Bledsoe & Barnes [19]  2003 | ✓ | ✕ | ✓ | N/A | N/A | N/A | N/A | N/A | ✓ | Valuable paper for critique of CISD and for providing alternatives. |
| Bracken-Scally et al. [49]  2015 | ✓ | ✓ | ✓ | ✓ | ✓ | ✕ | ✓ | ✓ | ✓ | Gives a broad overview of the implications for post-employment/retirement for those working as ESFR. |
| Chappell & Mayhew [28]  2009 | ✓ | ✓ | ✓ | ? | ✓ | ✕ | ✕ | ? | ✓ | Provides overview of the nature of violent incidents experienced by ambulance officers. However, little detail regarding their impact on ambulance officers’ mental health, as this component was explored using quantitative methods, but the sample size was too small for a statistical analysis to be conducted. |
| Clompus & Albarran [45]  2016 | ✓ | ✓ | ✓ | ✓ | ✓ | ✕ | ✓ | ✓ | ✓ | Aims to explore the resilience of paramedics and what toolkit of strategies they have to cope with workplace stressors. This toolkit reflects many of the strategies revealed in other papers. Defines them as formal strategies provided by the organisation, informal strategies of peers, etc., internal strategies of compartmentalising, and external strategies of seeking outside help. |
| Coxon et al. [46]  2016 | ✓ | ✓ | ✓ | ✓ | ✓ | ✓ | ✓ | ✓ | ✓ | Qualitative study interviews with dispatchers on how their job is perceived poorly by paramedics and the public. Little appreciation of the trauma and stress in managing to get paramedics to the sick person. Strongest focus was on need for training and education and for team building with paramedics. |
| Donnelly & Bennett [20]  2014 | ✓ | ✓ | ✓ | N/A | ✓ | N/A | N/A | ✓ | ✓ | Study developed a 27-point critical incident stress inventory based on one used for police, then modified and trialled. Qualitative component asked respondents to list additional critical incidents. Critical incidents were ordered according to number of times experienced and paramedic view on impact. |
| Donnelly & Siebert [9]  2009 | ✓ | ✓ | ✓ | N/A | ✓ | N/A | N/A | ✓ | ✓ | Provides a typology of stressors divided between those leading to PTSD and PTSS, then occupational and critical incident stressors. Coping mechanisms are divided into social (work place, leadership styles, organisation of service, culture of service as paramilitary). Personal is divided into psychological (locus of control), and demographic (years of service, gender, age, training levels). |
| Dropkin et al. [21]  2015 | ✓ | ✓ | ✓ | ✓ | ✓ | ✕ | ✓ | ✓ | ✓ | Focus is on physical health and injury in the job and the differences between management and on-road. |
| Flannery [22]  2015 | ✓ | N/A | ✓ | N/A | ? | N/A | N/A | ✓ | ✓ | The author demonstrates that single therapy interventions such as CBT have been shown to be ineffective for first responders, instead suggesting a multi-modal response by therapists to paramedic stress. |
| Forslund et al. [40]  2004 | ✓ | ✓ | ✓ | ✓ | ✓ | ? | ✓ | ✓ | ✓ | Paper focuses on call center operators about what is difficult and stressful in their job. |
| Gallagher & McGilloway [50]  2008 | ✓ | ✓ | ✓ | ✓ | ✓ | ✕ | ✕ | ✓ | ✓ | The findings suggest that exposure to CIs has a significant impact on health and well-being and identifies a range of barriers to support service utilisation. A need for professional counselling and stress awareness training was also identified. |
| Gist & Harris Taylor [23]  2008 | ✓ | N/A | ✓ | N/A | ✓ | N/A | N/A | ✓ | ✓ | Suggests core elements of psychological first aid. Recommends solid EAP services, not simply one-offs, but available to staff at their discretion and for issues wider than workplace. Provides a list of steps for assistance from the initial one of peer support, and the time for this to occur, to formal counselling. |
| Halpern et al. [34]  2009 | ✓ | ✓ | ✓ | ✓ | ✓ | ✓ | ✓ | ✓ | ✓ | Incidents identified as critical commonly involved patient death often combined with poignancy. They evoked vulnerable feelings of inability to help and intense compassion, which led to further emotional, cognitive, and behavioural responses. Difficulty in acknowledging distress and fear of stigma presented significant barriers to accessing support. These barriers may be overcome by educating both ambulance personnel and their supervisors to recognise and tolerate the vulnerable feelings often evoked by critical incidents. |
| Halpern et al. [35]  2009 | ✓ | ✓ | ✓ | ✓ | ✓ | ✕ | ✓ | ✓ | ✓ | Highlights not only the importance of supervisor support and a timeout period immediately following a critical incident, but also the barriers to these forms of support being provided. Of note, the interventions suggested or valued by participants differ from the traditional Critical Incident Stress Debriefing Format. |
| Hegg-Deloye et al. [36]  2014 | ✓ | N/A | ✓ | N/A | ✓ | N/A | N/A | ✓ | ✓ | The majority of studies included were quantitative. The authors identify areas for future research and the need for interventions, both at an individual and organisational level, to minimise emotional disorders among paramedics. |
| Hugelius et al. [41]  2014 | ✓ | ✓ | ✓ | ✓ | ✓ | ✕ | ✕ | ✕ | ✓ | Useful insight into crisis support interventions provided to personnel in Sweden. Larger sample size needed. Consensus reached by participants, indicating some transferability. Validity could be improved if analysis conducted on transcripts derived from audio-recordings rather than interview notes only. |
| Jonsson & Segesten [42]  2004 | ✓ | ✓ | ✓ | ✓ | ✓ | ✓ | ✓ | ✓ | ✓ | The findings provide a detailed description of the emotions experienced by ambulance personnel at all stages of a traumatic experience, from pre-arrival through to post-event. |
| Klimley et al. [24]  2018 | ✓ | N/A | ✓ | N/A | ✓ | N/A | N/A | N/A | ✓ | The majority of the articles included in the review that pertain to emergency medical dispatchers appear to be either quantitative in nature or were published prior to the year 2000, highlighting a need for further qualitative research into the mental health of this group of emergency service workers. |
| Lindahl [28]  2004 | ✓ | N/A | ✓ | N/A | N/A | N/A | N/A | N/A | N/A | No limitations identified by author. |
| Mahony [48]  2005 | ✕ | ✓ | ✓ | ? | ✓ | ✓ | ✕ | ? | ✓ | The article highlights the range of organisational stressors experienced by ambulance officers as a result of corporate restructuring, many of which arise from increased workload intensity. |
| Mahony [54]  2001 | ✓ | ✓ | ✓ | ? | ✓ | ✓ | ✕ | ? | ✓ | An in-depth exploration of occupational stressors experienced by ambulance personnel. It is acknowledged that stressors reported by participants were similar to those that could be found in other organisations. |
| Paterson et al. [10]  2014 | ✓ | ✓ | ✓ | ✓ | ✓ | ✕ | ? | ✓ | ✓ | Both organisational and non-organisational factors were identified by paramedics as causing fatigue. Further exploration using interviews or focus groups would allow participants to elaborate on their experiences. |
| Pow et al. [37]  2017 | ✓ | N/A | ✓ | ✓ | ✓ | ✕ | ✓ | ✓ | ✓ | Quantitative data provides useful information on beneficial impact of perceived support on sleep quality and minimising effects of occupational stress on sleep. The open-ended survey question provided an opportunity for participants to identify occupational stressors not already included in the survey instrument. |
| Pyper & Paterson [29]  2016 | ✓ | ? | ✓ | ✓ | ✓ | ✓ | ✓ | ✓ | ✓ | Predominantly quantitative, but with some relevance through the open question analysis. |
| Regehr & Millar [38]  2007 | ✓ | ✓ | ✓ | ✓ | ✓ | ? | ✓ | ✓ | ✓ | Provide a good basis for understanding paramedic challenges with managing organisational stress. |
| Regehr et al. [39]  2002 | ✓ | ✓ | ✓ | ✓ | ✓ | ✓ | ✓ | ✓ | ✓ | Outlines the sources and responses to workplace/organisational stress. |
| Rice et al. [30]  2014 | ✓ | ✓ | ✓ | ✓ | ✓ | ✓ | ✓ | ? | ✓ | Some relevance to the question of the relationship between stress and physical health/not predominantly paramedic-focused. |
| Roth & Moore [24]  2009 | ✓ | ✓ | ✓ | ✓ | ✓ | ✓ | ✓ | ✓ | ✓ | Provides insight into the relationship between family and those that work in emergency services and how the work affects the family system. |
| Sofianopoulos et al. [31]  2012 | ✓ | N/A | ✓ | N/A | ✓ | N/A | N/A | ✓ | ✓ | The review provides a good overview, and the findings suggest that further work is required to examine shift hours and workforce health and safety in the pre-hospital setting. |
| Varker et al. [32]  2018 | ✓ | N/A | ✓ | N/A | ✓ | N/A | N/A | ✓ | ✓ | An Evidence Map identifies local areas of need and the gaps. |
| Wiitavaaraa et al. [40]  2007 | ✓ | ✓ | ✓ | ✓ | ✓ | ✓ | ✓ | ✓ | ✓ | Valuable in terms of understanding how physical illness is perceived and conceptualised for ambulance personnel |
| Wolkow et al. [33]  2015 | ✓ | N/A | ✓ | N/A | ✓ | N/A | N/A | ✓ | ✓ | Extreme sleep restriction over multiple days of emergency work can disrupt the circadian cortisol rhythm, disrupt pro- and anti-inflammatory cytokine levels, elicit adverse psychological responses, and cause a simultaneous increase in both cortisol and cytokine levels. |

✓ - Yes, ✕ - No, ? – Could not be determined, N/A – Not applicable
