# Supplementary material for: The effects of emergency medical service work on the psychological, physical, and social well-being of ambulance personnel: a systematic review of qualitative research
Source: BMC Psychiatry. 2020 Jul 3;20:348. doi: 10.1186/s12888-020-02752-4 (PMC7332532; doi:10.1186/s12888-020-02752-4)
Supplement: Supplementary file 6 — Additional file 6: Appendix 6. CASP quality ratings of systematic review [file 12888_2020_2752_MOESM6_ESM.docx]

**Appendix 6: CASP quality ratings of systematic reviews**

| **Author (year)** | **Clearly focused question** | **Right type of papers** | **All the important, relevant studies included** | **Quality of included studies assessed adequately** | **Reasonable to combine the results** | **Overall results** | **Preciseness of results** | **Applicability of results to local population** | **All important outcomes considered** | **Benefits worth harms & costs** | **Comments** |
| --- | --- | --- | --- | --- | --- | --- | --- | --- | --- | --- | --- |
| Golding et al. [47]  2017 | ✓ | ✓ | ✓ | ✓ | ✓ | ✓ | ✓ | ✓ | ✓ | ✓ |  |
| Larsson et al. [43]  2016 | ✓ | ✓ | ✓ | ✓ | ✓ | ✓ | ✓ | ✓ | ✓ | ✓ | Clearly reported systematic method and thematic analysis of results. |
| Skogstad et al. [51]  2013 | ✓ | ✓ | ✓ | ✓ | ✓ | ✓ | . ✓ | ? | ✓ | ✓ | Clear systematic review with analysis of the key issues |
| Sterud et al. [11]  2006 | ✓ | ✓ | ✓ | ✓ | ✓ | ✓ | ✓ | ✓ | ✓ | N/A | Well-structured review with clear exclusion and inclusion criteria with critical analysis. Reported significant effects of work on ambulance personnel, but lack of consistency in the measures across studies. |

✓ - Yes, ✕ - No, ? – Could not be determined, N/A – Not applicable
